# Structure functional insights into calcium binding during the activation of coagulation factor XIII A

**DOI:** 10.1038/s41598-019-47815-z

**Published:** 2019-08-05

**Authors:** Sneha Singh, Johannes Dodt, Peter Volkers, Emma Hethershaw, Helen Philippou, Vytautus Ivaskevicius, Diana Imhof, Johannes Oldenburg, Arijit Biswas

**Affiliations:** 1 Institute of Experimental Hematology and Transfusion medicine, University Hospital of Bonn, Bonn, 53127 Germany; 2 Paul-Ehrlich Institute, Langen, 63225 Germany; 3 Discovery and Translational Science Department, University of Leeds, Leeds, LS29JT United Kingdom; 4 Pharmaceutical Biochemistry and Bioanalytics, Pharmaceutical Institute, University of Bonn, An der Immenburg 4, Bonn, 53121 Germany

**Keywords:** Blood proteins, Computational biology and bioinformatics

## Abstract

The dimeric FXIII-A_2_, a pro-transglutaminase is the catalytic part of the heterotetrameric coagulation FXIII-A_2_B_2_ complex that upon activation by calcium binding/thrombin cleavage covalently cross-links preformed fibrin clots protecting them from premature fibrinolysis. Our study characterizes the recently disclosed three calcium binding sites of FXIII-A concerning evolution, mutual crosstalk, thermodynamic activation profile, substrate binding, and interaction with other similarly charged ions. We demonstrate unique structural aspects within FXIII-A calcium binding sites that give rise to functional differences making FXIII unique from other transglutaminases. The first calcium binding site showed an antagonistic relationship towards the other two. The thermodynamic profile of calcium/thrombin-induced FXIII-A activation explains the role of bulk solvent in transitioning its zymogenic dimeric form to an activated monomeric form. We also explain the indirect effect of solvent ion concentration on FXIII-A activation. Our study suggests FXIII-A calcium binding sites could be putative pharmacologically targetable regions.

## Introduction

Calcium ions (Ca^2+^) play a major role in the tight regulation of coagulation cascade that is paramount in the maintenance of hemostasis^1,2^. Other than platelet activation, calcium ions are responsible for complete activation of several coagulation factors, including coagulation Factor XIII (FXIII)^3^. FXIII is responsible for covalently cross-linking preformed fibrin clots preventing their premature fibrinolysis, by maintaining the clot architecture and strength. FXIII circulates in plasma as a heterotetrameric pro-transglutaminase (pFXIII), complex FXIII-A_2_B_2_ composed of dimeric subunits of catalytic FXIII-A and protective/regulatory FXIII-B^4–7^. Although the catalytic FXIII-A subunit bears several structural and sequence similarities with other transglutaminases, it is also unique in specific aspects^8^. Unlike other members of the transglutaminase family, FXIII-A is the only molecule activated by a combination of calcium binding and proteolytic thrombin cleavage of an N –terminal 37-amino acid region [activation peptide (FXIII-AP)]. Additionally, it is also the only transglutaminase whose functional molecule is a complex, unlike other transglutaminases that are monomeric. FXIII-A, like all other transglutaminases belongs to a category of calcium-binding proteins that lacks EF-hand motif, a typical structural helix-loop-helix topology commonly found in calcium binding proteins^9^. Transglutaminases are believed to have evolved from an ancient cysteine protease in bacteria^10^. The microbial transglutaminase though has evolved divergently from the eukaryotic transglutaminases^11^. Although calcium-binding is common to the activation pathway of all transglutaminases, different members of the transglutaminase family show additional regulatory/functional features in their activation mechanisms, despite sharing a structural and sequential similarity. For instance, Transglutaminase 2 (TG2) has multiple regulatory features in addition to calcium binding like vicinal disulfide bonds and GTP/GDP binding which in combination control the activity status of the protein at different ionic conditions^9^. The FXIII-A subunit is a structurally well-characterized protein with several zymogenic crystal structures, in ion-bound (to different cations; PDB IDs: 1ggu, 1ggy, 1qrk) and unbound states (PDB ID: 1fie, 1f13) present in the protein structure database^12–14^. The recent disclosure of the non-proteolytically activated form of FXIII-A (FXIII-A*) (PDB ID: 4kty) has shown the presence of three calcium binding sites in FXIII-A^15^. The first FXIII-A calcium binding site (Cab1) involves the residues Ala457, Asn 436, Glu 485 and Glu 490, the second calcium binding site (Cab2) uses the residues Asp 351, Gln 459, Asn 347, Asp 349, and Asp 343 and the third calcium binding site (Cab3) coordinates the residues Asp 271, Asn 267, Asp 270 and Ala 264^15^. In our earlier study, while explaining the concerted model of FXIII activation we had demonstrated *in silico* that the first calcium binding site (Cab1) that is usually observed constitutively coordinated in specific zymogenic FXIII-A crystal structures (E.g. PDB ID: 1ggu), actually lays a stabilizing influence on the zymogenic form^12,16^. We also showed that its transient disruption upon thrombin-mediated FXIII-AP cleavage and simultaneous coordination of the other two calcium binding sites (Cab2, Cab3) is essential for the conformational activation of zymogenic FXIII-A. Cellular FXIII (cFXIII) that is inaccessible to plasma thrombin requires high supra-physiological levels of calcium or a combination of high sodium and physiological calcium for non-proteolytic activation. Hence, Cab1 as a zymogenic constraint acts antagonistically to the other two calcium binding sites keeping the molecule inactive intracellularly in the absence of thrombin unless provoked by high ion concentrations^16^.

In continuation of our preliminary work and observations, our current study takes a deeper look into the calcium binding sites of FXIII-A. We investigate how these sites contribute to the functional evolution of FXIII molecule as a unique protein in the transglutaminase family. We characterize the three FXIII-A calcium binding sites concerning their evolution, mutual crosstalk, and effect on FXIII-A thermodynamic profile, substrate binding and interaction with other similarly charged ions. The FXIII-A calcium binding sites show a high degree of conservation within transglutaminases, but at the same time, they also possess unique spatial and structural features that differentiate FXIII-A as a unique molecule amongst transglutaminases. Our results confirm that saturation of the first calcium-binding site lays a zymogenic constraint that resists the activation of FXIII-A, as was hypothesized in our earlier work^16^. The thermodynamic profile of FXIII-A activation observed during increased calcium binding reiterates recent observations that calcium binding to FXIII-A results in major conformational changes leading up to the dissociation of the FXIII-A_2_ dimer during activation to a monomeric activated FXIII-A (FXIII-A*) form. Using all atoms MD simulations of FXIII-A and its core domain under different ionic concentrations we show that apart from coordination with charged residues, the mere presence of cations can also bring about subtle changes in the structure of FXIII-A by altering the surface electrostatics. The alteration of FXIII-A subunit´s surface electrostatics can, in the long term, influence the activation status of the molecule in different ionic conditions.

## Results

### Calcium binding site residues on FXIII-A molecule are highly conserved

**Figure 1:**
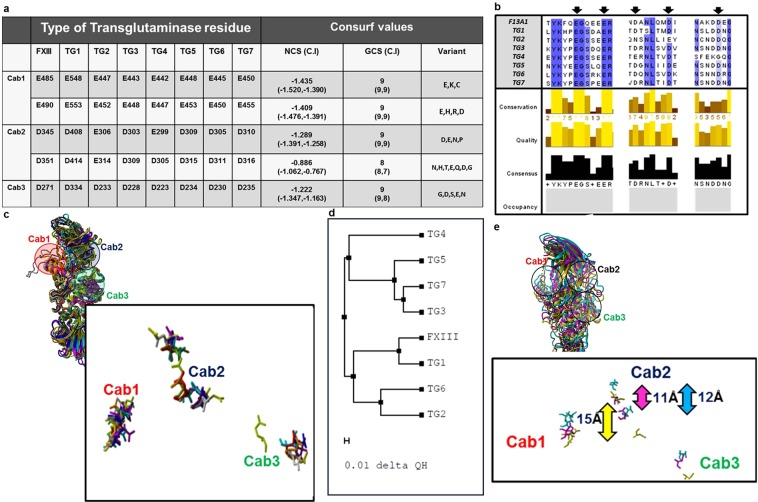
Conservation of calcium binding sites in Transglutaminases with respect to FXIII-A. Panel a tabulates the conservation results generated for the FXIII-A amino acid sequence input (Uniprot ID: P00488; F13A_HUMAN) on the *Consurf* server. Abbreviations: NCS: Normalized conservation score, GCS: Generalized conservation score. Panel b illustrates the actual alignment generated as output on the *Consurf* server for the FXIII-A subunit sequence input. The highly conserved residues are shaded in blue. Arrows indicate the binding site residues mutated in this study. Panel c shows a structural alignment of all seven human Transglutaminases i.e. of three crystal structures TG2, TG3 and FXIII-A and threaded models for the remaining four transglutaminases. The structures are depicted in ribbon format. The inset image shows the spatial alignment of the calcium binding site residues of all transglutaminases as stick models. The different transglutaminases are colored differently. Panel d shows a structural similarity tree generated from the structural alignment on Panel c. The distances on the tree represented by the delta QH value is proportional to the dissimilarity between two structures i.e. the farther two structures are there on the tree, the more structurally unlike they are. Panel e shows the structural alignment of only three human Transglutaminases with biophysical crystal structures i.e. TG2, TG3 and FXIII-A. The inset diagram the spatial alignment of the calcium binding site residues as stick models of the three human transglutaminases. The structures are depicted in ribbon format with TG2, TG3 and FXIII colored yellow, magenta and cyan respectively. The bidirectional arrows represent the C-α backbone atom distances between the D345 and D351 residue (FXIII; cyan), E306 and E314 residue (TG2; yellow), and D303 and D309 residue (TG3; magenta).

Within the multiple alignments of FXIII-A with other known transglutaminases, all three calcium binding site residues showed a high degree of conservation [Average generalized conservation score: 9 (range = 8–9)] (Fig. 1a). However, several variant substitutions observed in the multiple alignments corresponding to these residues, including few (Cab1 residues *E485* and *E490*) in which the substitution was to an oppositely charged residue. These residues occur in a semi-conserved region with the conservation status varying between high to low conservation between alternating residues (Fig. 1b). The structural alignment of the crystal structures of FXIII-A, TG2, TG3 and threaded models of TG1, TG4, TG5, TG6, TG7 shows proper domain-wise alignment with the average RMSD of 1.2 Å (Range: 0.5–1.71 Å) (Fig. 1c). FXIII-A shows the closest structural similarity with TG1 (0.5 Å RMSD) (Fig. 1c,d). The three calcium binding sites were in spatially similar locations in the aligned structures (Fig. 1c). However, the distance between their backbone atoms as well as the orientation of the side chain residues differed between transglutaminases (Fig. 1e).

### Biochemical endpoint FXIII activity assays reveal that calcium binding influences the formation of substrate binding pockets on FXIII-A molecule

**Figure 2:**
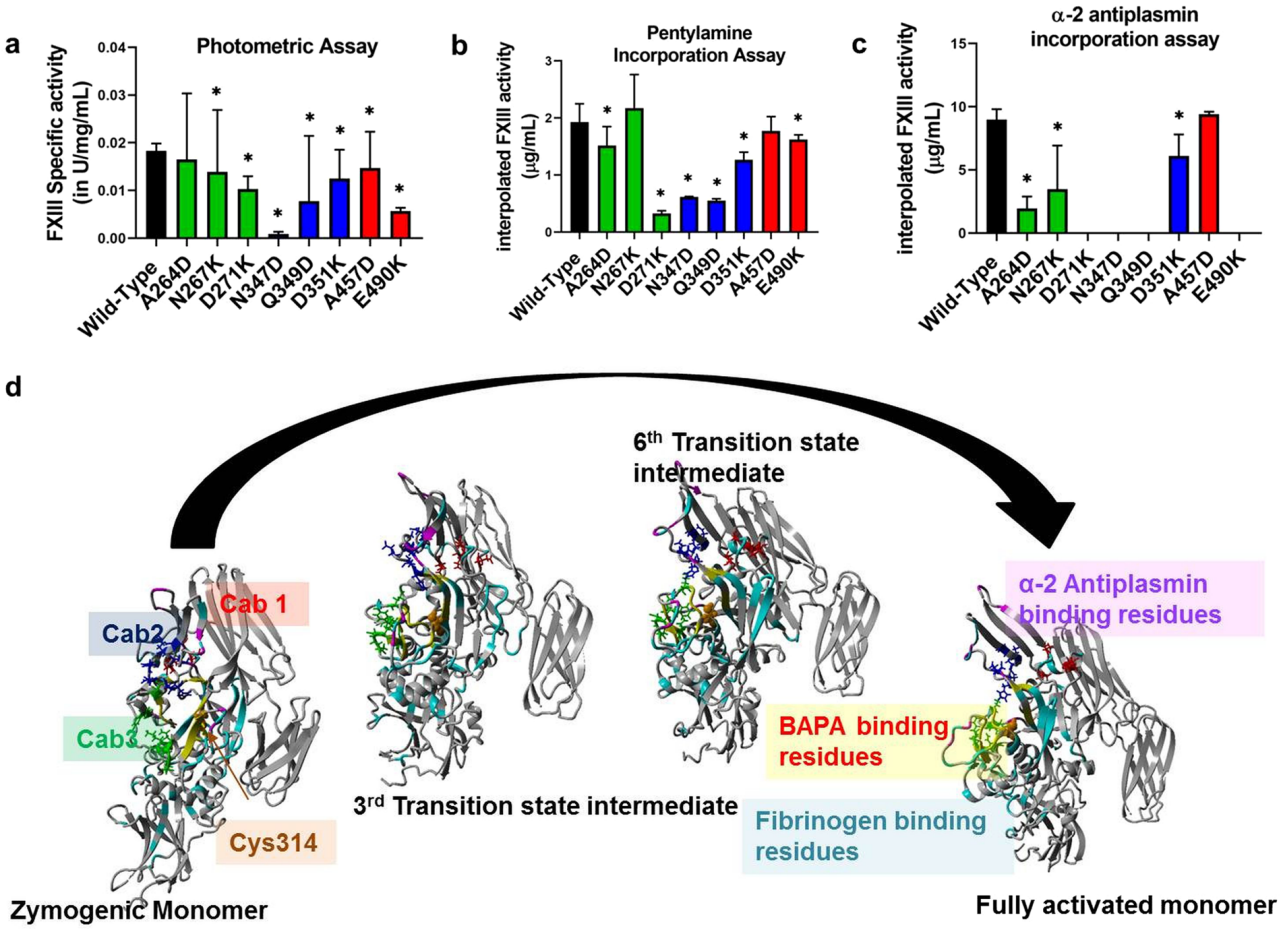
Effect of rFXIII-A calcium binding site mutants on its specific activity and substrate specificity. Panels a, b and c represent comparative bar graphs for Specific activity of intracellular lysates (wild type and variant) based on Photometric assay, interpolated FXIII-A activity based on Pentylamine incorporation assay and α-2 antiplasmin incorporation assay, respectively. In the event a variant is significantly different than the wild type, it is marked with a star (*) sign on top of the corresponding bar. Statistical significance is set at p < 0.05. Experiments were performed in duplicates, thrice (N = 6). Panel d shows the putative fibrinogen, α-2 antiplasmin and BAPA binding site residues on zymogenic FXIII-A monomeric and activated monomeric FXIII-A* structures along with two intermediate transition state structures. The structure backbone is depicted in grey colored ribbon format. The putative binding site region backbones are colored cyan for fibrinogen, yellow for BAPA and magenta for α-2 antiplasmin. The three calcium binding sites are also depicted on all four structures as stick models colored red (Cab1), blue (Cab2) and green (Cab3) respectively.

Biochemical endpoint FXIII activity assays, which primarily evaluate transglutaminase cross-linking function when evaluated for the FXIII-A calcium binding mutants showed most of them lacking in cross-linking ability. The photometric assay, combined with antigenic determination revealed that the specific activity of FXIII-A was reduced in all mutants expressed when compared with the wild type (0.02 ± 0.001 IU/mg/mL). The mutant N347D reported the lowest specific activity (0.00087 ± 0.0001 IU/mg/mL; p < 0.0001) (Fig. 2a). Pentylamine incorporation assay also reported low levels of interpolated FXIII-A activity (incorporated/crosslinked substrate in µg/mL) in all mutants except N267K (2.17 ± 0.58 µg/ml) in which it was non-significantly elevated when compared to the Wild type (1.92 ± 0.31 µg/mL; p = 0.42) (Fig. 2b). The lowest interpolated FXIII-A concentration was observed for the mutant D271K (0.32 ± 0.04 µg/mL; p < 0.0001). Similar to the Pentylamine incorporation assay, average interpolated active FXIII-A* concentration from the α-2-antiplasmin incorporation assay using fibrin as a substrate (in µg/mL) was also reduced for all mutants when compared with the wild type (8.97 ± 0.83 µg/mL), except in mutant A457D which showed non-significantly elevated levels (9.40 ± 0.02 µg/mL; p = 0.09) (Fig. 2c). One mutation from the Cab3 (D271K) and two from Cab2 (N347D, Q349D) showed no detectable α-2-antiplasmin incorporation (i.e. <1.25 µg/mL). The putative substrate binding regions corresponding to fibrinogen substrate (in cyan) outnumber those of substrates α-2-antiplasmin (in magenta) and BAPA [5-(Biotinamido) pentylamine; PubChem ID: CID 83906] (in yellow) (Fig. 2d). This difference in putative binding sites is expected considering the relative sizes of these substrates, fibrinogen being the largest and BAPA the smallest (Supplementary Fig. 1). While some of these regions lie close to the active site, others are far from it and most likely have an allosteric role in substrate binding. Interestingly, fibrinogen substrate binding regions cover almost all calcium binding sites, but those of α-2-antiplasmin appear closer to Cab2 while those of BAPA lie closer to Cab3 (Fig. 2d).

### Real-time monitoring of FXIII-A* generation confirms that Cab1 is a zymogenic constraint on FXIII-A

**Figure 3:**
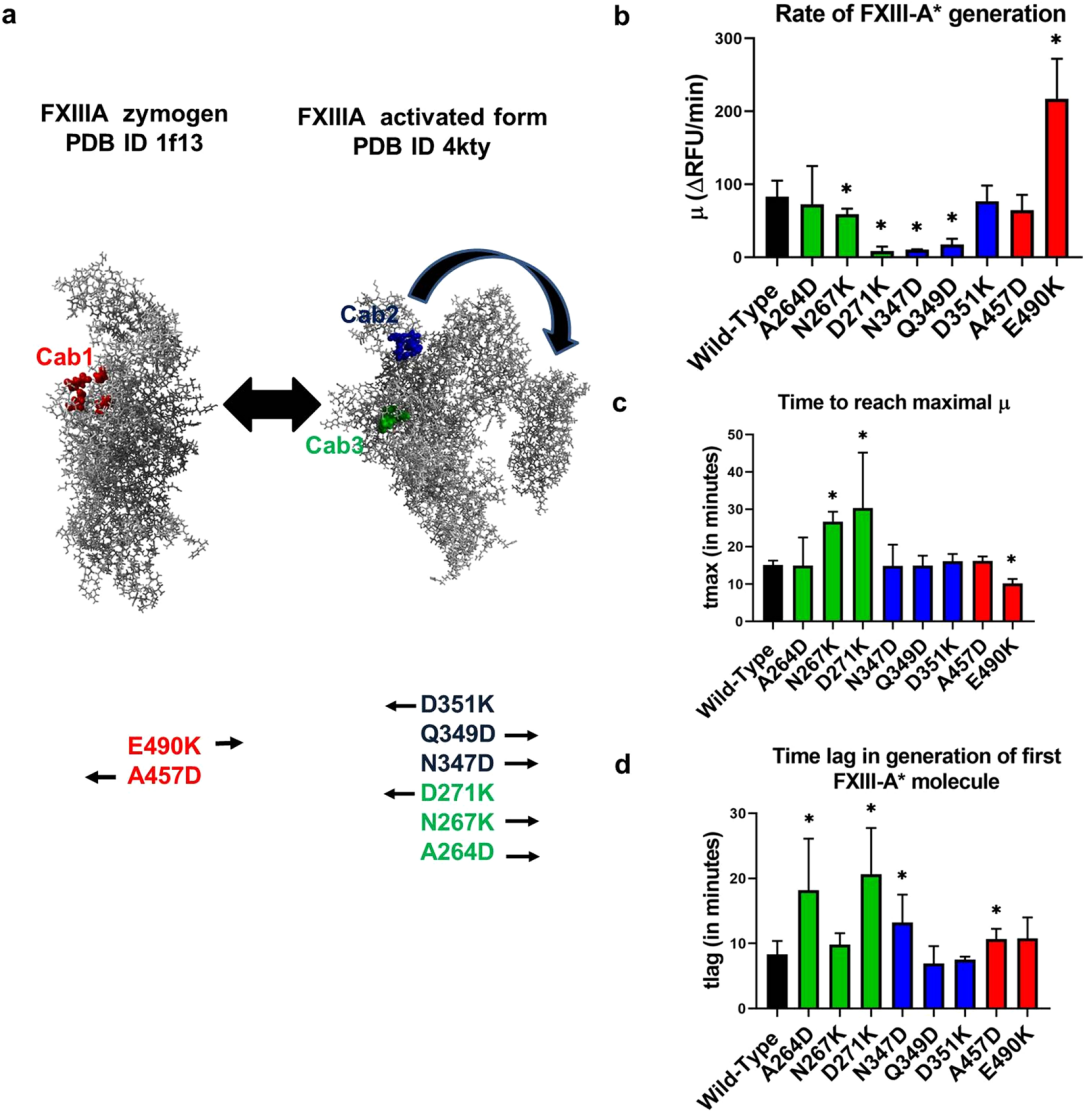
Results of FXIII-A* generation assay for rFXIII-A calcium binding site mutants. Panel a explains the principle underlying different rFXIII-A calcium binding site mutants generated in this study. It depicts the conformational transformation between the zymogenic and activated FXIII-A crystal structures. Both structures are depicted as grey ball and stick models. The first calcium binding site (Cab1) residues are depicted as red ball models on the zymogenic FXIII-A crystal structure because the coordination of this site is a zymogenic constraint. The second and third calcium binding site (Cab2 and Cab3) are depicted as blue and green ball models respectively on the activated FXIII-A* crystal structure. Below both these structures, the mutants generated for this study are listed with arrows indicating the anticipated structure favored by each of this mutant as per our hypothesis. Panels b, c and d depict the comparative bar graphs corresponding to three major parameters (μ, tmax and tlag) obtained from the FXIII-A* generation assay. In the event a variant is significantly different than the wild type, it is marked with a star (*) sign on top of the corresponding bar. Statistical significance is set at p < 0.05. Experiments were performed in in duplicates, thrice (N = 6). Please note, FXIII-A* generation data for the mutants D271K and N347D show very low activity and a fit of the data to the model revealed no valid estimates.

The FXIII-A* generation assay which tracks the rate of FXIII-A* generation real-time reported differences from the endpoint assay for the calcium binding site mutants in several of the parameters derived from its curve (Fig. 3, Supplementary Fig. 2). The calcium binding site mutations have been designed keeping in mind our earlier formulated and reported hypothesis that making the Cab1 more positive will result in acceleration of FXIII-A activation while doing the same to Cab2 and Cab3 will impede activation and vice-versa (Fig. 3a)^16^. The Cab1 mutant E490K reported an almost ~2.5x times higher rate of FXIII-A* generation (µ) (217.36 ± 54.74 R.F.U./min) than the wild type rFXIII-A (83.17 ± 21.88 R.F.U./min) (Fig. 3b). The other Cab1 mutant A457D reported a lower rate of FXIII-A* generation but not significantly, so (64.34 ± 21.36 R.F.U./min). One mutation from the Cab3 (D271K) and two from Cab2 (N347D, Q349D) revealed a highly significant decrease in the rate of FXIII-A* generation/activation (Fig. 3b). The parameter tmax (time taken to reach the maximal rate µ) revealed a good inverse correlation with the rate of FXIII-A* generation or µ, with the lowest value for tmax consistent with the highest µ observed for Cab1 mutant E490K (Fig. 3c). The parameter tlag (time required for the activation of FXIII-A by thrombin) was elevated for all mutants when compared to the wild type rFXIII-A except for the Cab2 mutant Q349D, which reported lower but non-significant values (6.90 ± 2.69 min) (Fig. 3d). The thrombin resistant mutant FXIII-A-R38A showed an exponential increase in the rate of FXIII-A* generation when the assay was performed with increasing levels of calcium concentration (Supplementary Fig. 3). However, it is noticeable that the exponential trend-line is a better fit at levels above 20 mM calcium, suggesting that this concentration might be rate-limiting for non-proteolytic activation of FXIII-A. The maximal rate of FXIII-A* generation of the corresponding standard plasma was observed to be 158.8 R.F.U./min.

### Simulation of FXIII-A at different ionic concentrations reveals that ancillary ions play a role in bringing the molecule to a pre-activated state by altering surface electrostatics

**Figure 4:**
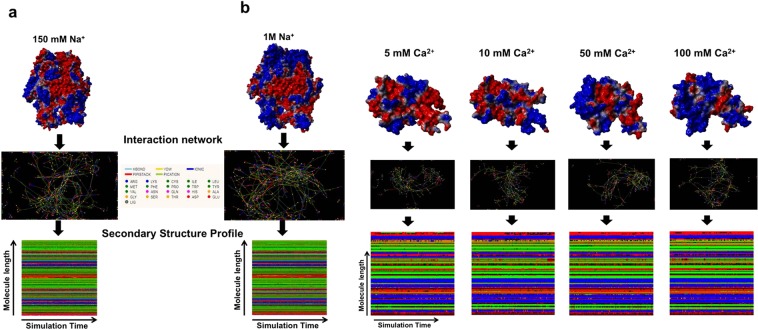
Effect of calcium and sodium on the structure of FXIII-A. Upper parts of panels a and b represent the simulation snapshots of full length FXIII-A molecule and FXIII-A core domain post 100 ns of simulation in different sodium and calcium concentrations respectively as indicated. The simulation snapshot structures are depicted by their molecular surface view with the electrostatic potential (Red: Negative, Blue: Positive) superimposed on it. Below the simulation snapshots in both panels are the Residue interaction network (RIN) or the inter-residue interaction chart extracted from the Ring 2.0 server by submitting to it the post 100 ns simulation snapshot as a PDB file under default server conditions. Lowermost section of both panels is the Secondary structure profile for the entire 100 ns of simulation plotted for the entire length of the molecule. The colour code for the secondary structure profile is the following: Coil: Red, Strand: Green, Helix: Blue, Turn: Black.

Molecular dynamic simulations of FXIII-A and its core domain performed under different concentrations of calcium and sodium shed some light into the activation behavior of FXIII-A when exposed to binding (calcium) as well as non-binding (ancillary; sodium) ions. Simulations for the full zymogenic FXIII-A crystal structure (source PDB ID: 1f13) equilibrated close to the 30 ns time point between 1–1.5 Å RMSDs for both physiological and high sodium ion concentrations. The core domain simulations, on the other hand, equilibrated faster closer to 20 ns and between 1.5–3.5 Å RMSDs, depending on the calcium ion concentration of the simulation (Supplementary Fig. 4). All simulation trajectories in the production phase i.e., only post equilibrations were considered relevant and analyzed. Simulations performed at different ion concentrations for both the full zymogenic FXIII-A subunit as well as the core domain did not demonstrate any drastic changes in overall structure post equilibration (Fig. 4a). The lack of any significant differences in overall structure is expected owing to the relative short time scale of simulation (100 ns; consistent conformational changes on a large scale i.e., >5 Å will require runs close to µseconds which considering the size of our simulations, i.e., 85,000–500,000 atoms would be computationally too expensive for this study). However, a noticeable change in the surface electrostatic pattern/distribution was observed (Fig. 4b). Increasing cation concentrations in different simulations resulted in an increasing spread of positive potential over the surface of the simulated structures (Fig. 4b). This change was also coupled to change in RINs within these structures (Fig. 4a,b). The overall impact was that the changes in RINs within these structures were bringing about a change in their secondary structure profile. However, the changes in the secondary structure profile were very subtle and not amply visible on the secondary structure profile, most likely owing to the shortness of the simulation runs.

### Thermodynamic analyses of calcium binding to FXIII-A suggests that FXIII-A activation is a stepwise process resulting in its monomerization upon activation

**Figure 5:**
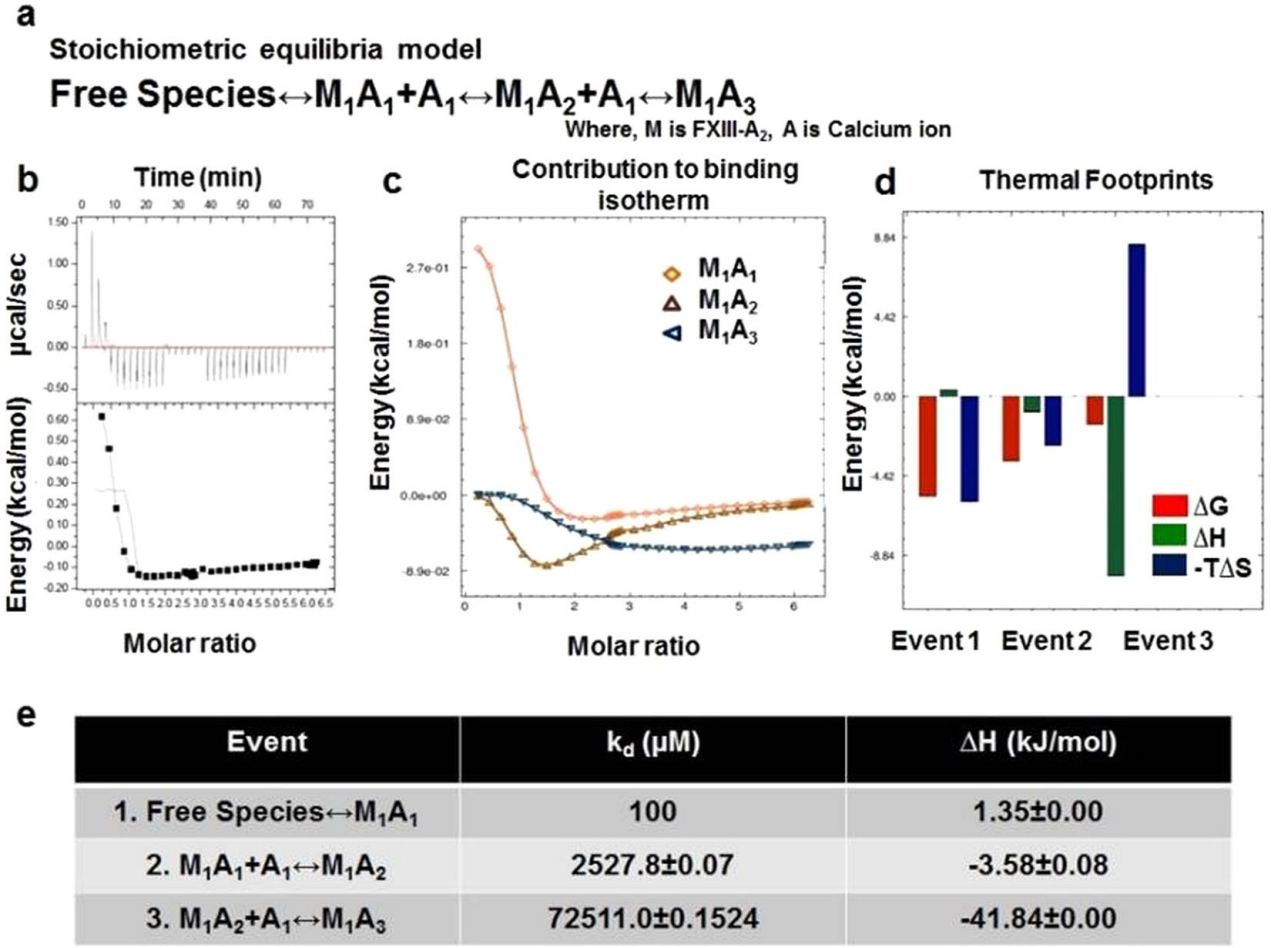
Calcium binding to rFXIII-A_2_ studied by ITC. Panel a. Equation depicting the stoichiometric binding equilibrium model, followed for the analysis of data derived from ITC (Model was generated in *Affinimeter* using model builder approach). Panel b. Titration of 1.25 mM rFXIII-A_2,_ with 25 mM CaCl_2_ (c-value = 20). Upper image of this panel is the raw data depicting the heat change upon each injection; lower image in this panel is the normalized data, with integrated heat change plotted against the concentration ratio of calcium vs rFXIII-A_2._ Solid black line represents the corresponding fit obtained in Origin software using sequential binding mode, with n = 3. All heat changes are plotted after subtraction of reference titration (no rFXIII-A_2_ in sample cell vs. 25 mM CaCl_2_ in the presence of thrombin, at same conditions) Panels c, d and e are based on evaluation performed on *Affinimeter* (see Supplementary Fig. 5 for corresponding fit curves). Panel c depicts the contribution of individual species, generated during a sequential binding of calcium to FXIII-A (based on earlier observations^19^ and as participants of the Stoichiometric equilibria here (Panel A)), towards the binding isotherm. Panel d is the comparative heat signatures, or the thermal footprints obtained at each binding event following the equation ∆G = ∆H - T∆S (2nd Law of Thermodynamics); explaining the three thermodynamic events, and their corresponding changes in free energy (∆G), entropy (-T∆S) and enthalpy (∆H) (for the corresponding data obtained at c-value = 25,000 please refer to the Supplementary Fig. 6). Panel e is table representing event-wise changes in enthalpy (∆H), in kJ/mol, and corresponding change in binding affinity of calcium ions towards FXIII-A in all three corresponding events as per the stoichiometric equilibria model.

Our ITC thermodynamic profiles suggest that at varying c-values [i.e., ratio of the concentration of ligand in the injection (calcium) to the concentration of macromolecule in the cell (rFXIII-A_2_)]; the binding profile of calcium to rFXIII-A_2_ is different. Our thermodynamic profiles reveal that the binding of calcium is not a simple protein-ligand association; rather, it is a complicated mechanism involving conformational changes coupled with domain movement and subunit dissociation taking place simultaneously. Amongst a range of c-values tested, a c-value of 20 yielded an interesting thermodynamic profile representative of all aspects of calcium binding and subsequent rFXIII-A activation (Fig. 5). Other c-values either yielded no binding or were low on informational content (Supplementary Figs 5 and 6, for c-value 25,000). Fitting exercises performed in *Affinimeter*, following a custom model binding approach (see Method section). The thermodynamic activation profile is divided into three events. The titrations involving c-value = 20 (1.25 mM rFXIII-A_2_ (sample cell) and 25 mM calcium (syringe: CaCl_2_)), showed endothermic values with ∆H = 1.35 kJ/mol for the first binding event (M1 + A1 ↔ M1A1,k_D1_ 100 µM)^17^. In the second binding event (M1A1 + A1 ↔ M1A2, *k*_*D2*_ 2.7 mM), calcium binding affinity was lower with ∆H = −3.58 ± 0.08 kJ/mol (exothermic). The third and final binding event (M1A2 + A1 ↔ M1A3, *k*_*D3*_ = 72.5 mM), calcium affinity was also low with ∆H = −41.84 kJ/mol (exothermic) (Global- χ^2^ = 0.11). The overall analysis suggests that all three reaction components involve entropy changes (see thermal footprints, Fig. 5d) with ∆∆S (change in entropy) getting more positive with the binding event taking place. All the three events were spontaneous (∆G < 0) at given conditions (temperature T = 30 °C). The first event involving the saturation of Cab2 and transient disruption of Cab1 following its binding is an entropy-driven event. The second event involving saturation of Cab2 and solvent exposure of the dimeric interface is also driven by entropy. The third event i.e., the saturation of Cab3 resulting in dimer disruption, is an enthalpy-driven event different than the first two events. The contribution of individual species towards the thermogram (Fig. 5c), suggests that the intermediate M1A1 (Cab1 saturated rFXIII-A_2_ with FXIII-AP cleaved), is high energy unstable intermediate which undergoes heat absorption (endothermic). For data evaluation, a proper fit was achieved in *Affinimeter*. The data fitting depicted in Fig. 5b represents the raw data and fitting based on the sequential binding site model of Origin software (with fixed parameter K_d_ and ∆H) (also see Supplementary Fig. 5, for fitting curves obtained from *Affinimeter*). Blank measurements were made for thrombin and FXIII-A in the cell, against buffer to rule out the heat changes due to ion hydration. The absence of any heat changes in the blank ITC experiment overrides the possibility that the interpretations made here may be an effect of the heat of dilution. The data obtained after fitting exercises in *Affinimeter* following the stoichiometric equilibria model suggests significant enthalpy-entropy compensation involved in the binding of calcium to rFXIII-A_2_, to reach full saturation (thermal footprints, Fig. 5d, range of ∆∆G is much smaller than their associated changes in ∆∆H & ∆∆S)^18,19^.

## Discussion

### The structural and functional conservation of calcium binding sites within the transglutaminase family

Allosteric changes resulting from ion coordination of amino acid side chains often have fascinating functional implications^20^. Such coordination when they result in changes crucial to the catalytic cycle of a protein becomes a conserved trait across the evolutionary chain. The binding of calcium to FXIII and its paralogues and orthologues across the phylogenetic tree is a property that not only initiates the events of their catalytic cycle but in effect is responsible for all downstream changes. That includes dictating the rate of generation of the activated species, exposure of the catalytic triad, to the formation of substrate binding pockets and eventually (in the case of FXIII) formation of a monomeric activated form^16,21^. A closer look into the multiple sequence alignment of calcium binding site residues reveals that the negatively charged amino acids capable of side chain coordination are highly conserved (Fig. 1a). These residues usually occur in a neighborhood cluster of similarly charged amino acids which offsets the potentially deleterious effect of any unfavorable substitution at the coordinated residue by overtaking its coordination function (Fig. 1b). Therefore in spite of the high degree of conservation amongst calcium binding site residue, substituted variants including substitution to positively charged residues are observed in the conservation-related multiple sequence alignment. The existing literature indicates that amongst transglutaminases (EC 2.3.2.13) there can be as many as six high to low-affinity calcium binding sites. However, multiple alignments of a fairly large cohort of non-redundant sequences across the transglutaminase enzyme family indicate that at least in eukaryotic transglutaminases, three of these are of almost universal occurrence^22^. Unavailability of detailed structural information and the evolution of a compensatory regulatory motif might be the reason why some of these sites are not yet reported or not present in a few members of transglutaminase (TGase) family^23^. Amongst the structurally best-characterized transglutaminases, i.e., TG2, FXIII-A, and TG3; TG2 has additional regulatory mechanism^23^. These are the GTP/GDP switch (effective intracellular, at low calcium levels) and the vicinal disulfide bonds (effective extracellular, at high calcium levels). These additional regulatory mechanisms explain the need of six calcium binding sites in TG2 to induce the conformational pull needed for overall activation of the molecule (which therefore would be higher than FXIII or TG3). The enzymes, FXIII and TG3 differ from TG2 concerning calcium binding (Fig. 1c) because unlike TG2, both proteins have one constitutively bound calcium which confers their zymogenic forms stability while the other two sites are structurally shielded in the zymogenic state till they are functionally required. The TG3 is an inactive 77 kDa zymogen that must be cleaved into a 50 kDa N-terminal fragment containing the active site and a 27 kDa C-terminal fragment, which remains associated with the mature enzyme^24^. The zymogen binds a single calcium ion with high affinity (K_d_ = 0.3 µM)^25^. Upon cleavage, two additional ion binding sites with an average dissociation constant of 4 μM become available. Calcium coordination by Asp324 induces the movement of a loop region, enabling substrate access to the active site. The FXIII-A molecule, similar to TG1, has been reported in certain crystal structures to be bound constitutively to a single calcium ion at Cab1^12^. The activation events initiated by thrombin-mediated cleavage of its N-terminal activation peptide expose the other two calcium binding sites to coordinate, resulting in a series of conformational changes involving the formation of substrate binding pockets and ultimately exposing the catalytic triad^16^. Therefore, while retaining these three highly conserved calcium binding sites, these enzymes have functionally evolved differently to use these sites in a way that is best suited to their physiological milieu. Amongst FXIII-A and TG3, the one calcium binding site that imparts the zymogenic restraint differs. Sequence-wise and structurally, the constitutively bound zymogenic calcium binding site in FXIII-A (Cab1) effectively aligns with the activating/regulatory calcium binding site in TG3 (Cab3 in TG3) and vice versa. The spatial position of all three calcium binding sites in all transglutaminase structures is similar (Fig. 1c). However, the orientation of the binding site residue side chains varies along with their inter-atomic distance, between transglutaminases. As an example, the distance between the C-α backbone atoms of the Cab2 binding site residues (FXIII-A calcium binding site nomenclature) is shown in Fig. 1e. The C-α backbone inter-atomic distances are similar for TG3 and FXIII-A (11 and 12 Å respectively) with TG2 (15 Å) being different. The difference in inter-atomic distances is reflected in the similarity of the coordination pattern, as mentioned earlier for these proteins. Based on a structural relationship tree, we find that TG1 is more similar to FXIII-A than any other transglutaminase, which also is true evolutionarily since these two proteins are closely related (Fig. 1d). The overall structural similarity is reflected in similarity in calcium binding sites as well since the binding site residues align better between FXIII-A and TG1 than with TG7 which is far away on the structural tree from FXIII (Fig. 1d; Supplementary Fig. 7). Transglutaminases at a prokaryotic level have evolved from ancient cysteine proteases (*papain-like thiol proteases*); and several microbial transglutaminases, as well as cysteine proteases, show a dependency on calcium for the regulation of activity. Therefore one might assume that the mammalian FXIII calcium-binding sites have evolved from ancestral calcium binding sites in microbial transglutaminases and cysteine proteases^22^. However, microbial transglutaminases themselves have evolved divergently to eukaryotic transglutaminases. The only structurally fully characterized primitive transglutaminase from *Streptoverticllium mobaraense* (PDB ID: 1iu4) does not even show calcium dependence. Also the structural alignment of microbial cysteine protease that do show calcium dependence/binding like LapG (*Legionella pneumophila*) with the core domain of FXIII-A subunit shows no similarity in the spatial location of the calcium binding sites, even though the two structures align well with respect to sharing the conserved 4-sheet one-helix fold (Supplementary Fig. 8) of transglutaminases. All the above observations lead us to conclude that calcium binding sites in eukaryotic transglutaminases including FXIII have evolved divergently and have no evolutionary connection to the calcium binding sites in microbial transglutaminases or ancestral cysteine proteases.

### Cross-talk of FXIII calcium binding sites and their importance in the development of substrate binding pockets

In our earlier *in silico* study, we had presented the possibility that coordination of Cab1 of FXIII-A molecule stabilizes the zymogenic form of FXIII-A^16^. Our results from the generation assay support our hypothesis that the Cab1 binding site is a zymogenic constraint and that its transient disruption caused by calcium coordination at Cab2 post thrombin-mediated FXIII-AP cleavage is the rate determining step for FXIII-A activation (Fig. 3). The unique role of Cab1 is confirmed by the accelerated rate of activation of the molecule upon disrupting the calcium coordinating shells of Cab1 (E490K), disengaging calcium binding to Cab1, in the presence of an intact Cab2 (Fig. 3). The mutation (E490K) which introduces an additional positive charge within Cab1 has the same effect as the coordination at Cab2 i.e., it prevents the calcium binding at Cab1 thereby releasing this variant molecule from the zymogenic constraint imposed by Cab1, and it is then reflected in a significantly higher rate of FXIII-A* generation than the wild type as well as the other Cab variants (Fig. 3b). The endpoint assays, however, record lower activity of this mutant compared to the wild type (Fig. 2a–c), which re-emphasizes the fact that the rate of activation of the enzyme and the direct substrate turn-over are independent properties of the FXIII mediated catalysis especially when it comes to mutations/variants close to substrate binding or active site. The strong differences between the generation assay parameters and the endpoint assays (like photometric assay) indicate that the isopeptidase activity on which the generation assay is based^26^ and the transglutaminase crosslinking activity should not be considered synonymous especially when dealing with mutants. Our study is in close agreement with an earlier report that suggests that by mutating hydrophobic residues around the active site it is possible to have transglutaminase variants that are deficient in crosslinking activity but have normal or raised isopeptidase activity^24^. Certain calcium binding mutants (like E490K) therefore influence cross-linking activity negatively by altering the substrate binding sites of the acceptor or donor (cross-linking activity as also mentioned in the methods section is a two-step ping pong reaction). However, this same mutation shows a raised isopeptidase activity because by releasing the zymogenic constraint (of Cab1) the one-step conversion of FXIII-A to FXIII-A* (and the isopeptidase cleavage since no acceptor-donor cross-linked intermediate formation is required in this type of reaction) is facilitated. These observations present an interesting pharmaceutical possibility of engineering enzymes with multiple but quantitatively varying enzymatic capacities. Since FXIII-A possesses a transglutaminase, isopeptidase and also protein disulfide isomerase activity, it can serve as a model protein to generate variants with higher enzymatic efficiency than normal in one aspect but neutralized concerning the other activities^27,28^. A higher tlag observed for some calcium-binding mutants in the FXIII-A* generation assay is a representation of slow generation of the first active FXIII-A* species that can be attributed not just to thrombin cleavage but also to the underlying events which are responsible for the accessibility of active site cysteine (opening of molecule) for incoming substrate molecule (Fig. 3c). Therefore, the calcium binding site residues critically govern the structural integrity of the core domain with a possible distal allosteric effect on FXIII-AP. Whether these variants alter the binding affinity of FXIII-A to thrombin or directly impede the cleavage reaction cannot be ascertained from our data. The different endpoint-based assays between themselves revealed consistency only to a limited extent, i.e., all mutants reported consistently lower or similar activity status when compared to the wild type in all endpoint assays. However, quantitatively, these assays also revealed relative differences from one another for the mutants. For example, one mutation from Cab3 (D271K) and two from Cab2 (N347D, Q349D), reported consistently low levels in all these assays, indicating that the mutations distorted the substrate binding site (Fig. 2a–c). However, the extent of decrease in each assay was different, i.e., for the α2-antiplasmin assay all three mutants reported to have non-detectable levels, but for the other two assays variable detectable levels were observed which can be attributed to the fact that calcium binding sites spatially surround the catalytic site and are also proximal to the putative substrate binding sites. Their coordination subsequently plays a major role in the correct orientation of the substrate binding sites as can be seen in the transition state intermediate models^17^. Since the three assays are related to three different substrates, the relative impact on substrate affinity would depend on the extent to which each mutation affects the substrate binding site. As mentioned at the beginning of this paragraph, the disruption of Cab1 is also the rate-limiting step in FXIII-A activation which is highlighted by the increased response of the thrombin resistant FXIII-A subunit mutant (FXIII-A-R38A) in the generation assay to increased concentrations of calcium (Supplementary Fig. 3). The 20 mM calcium concentration appears to be the concentration barrier threshold above which calcium can coordinate Cab2 and Cab3 that eventually overpowers the zymogenic constraint associated with a coordinated Cab1. After the loss of the zymogenic constraint above this concentration, the rate of FXIII-A* generation follows an uninhibited exponential increase with increasing calcium concentrations (Supplementary Fig. 3).

### FXIII conformational changes during activation are effected at the secondary structure level and are governed by ion coordination and its surface electrostatic potential

**Figure 6:**
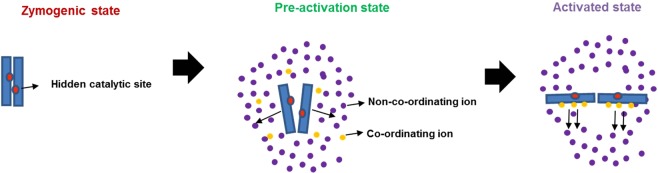
Effect of ancillary sodium ions towards generation of a pre-activation state of FXIII-A molecule. A cartoonist impression of the FXIII-A zymogenic state where no ions are influencing the structure followed by pre-activation state where the both the non-coordinating (assumed to be sodium in purple) as well as later coordinating ions (assumed to be calcium in yellow) influence the structure and conformation of FXIII-A molecule by altering its surface electrostatic properties. The last part of the figure depicts the fully activated molecule which is the final outcome of the coordination of the coordinating ions to its binding sites in a FXIII-A molecule that has already been moved from its zymogenic to a pre-activation state.

In several transglutaminases cross-talk between calcium and other ions (depending on the physiological milieu) influences the activity status of the protein. In TGM3, calcium ion coordination induces the movement of a loop region that enables substrate access to the active site. Tighter coordination with magnesium ion instead of calcium keeps the loop in its inactive configuration. Therefore, the relative concentrations of calcium and magnesium act as a regulatory switch for transglutaminase activity in TGM3^29^. FXIII-A protein which can be activated non-proteolytically by calcium only in the presence of supra-physiological levels of calcium (>50 mM) FXIII-A shows an interesting behavior in the presence of sodium (Fig. 4)^17^. In the presence of high levels of sodium (~1 M NaCl), the non-proteolytic activation requirement of calcium goes down to physiological levels (2.5 mM). The principle behind the effect of sodium on the non-proteolytic calcium-induced activation of FXIII-A is very much different from that observed for TGM3 in the case of magnesium and calcium since unlike in TGM3, where binding of both magnesium and calcium is observed, in FXIII-A there is no actual coordination of sodium. Our MD simulations performed on FXIII-A monomer at physiological versus high concentration (supra-physiological) of sodium ions in the simulation cell reveals that higher levels of sodium without actual binding (or coordination) to FXIII, alters the FXIII-A surface electrostatic distribution (Fig. 4a). This event most likely disturbs the pKa (ionization constant) of internal buried residues, thereby affecting intra-domain residue interactions and changing the local secondary structure, which results in subtle conformational changes. Very high concentrations of 1 M sodium likely alter the secondary structure of FXIII to the extent of overcoming the primary restraint induced by Cab1 bringing the molecule to the tipping point of activation but not activating it (Fig. 6). At this point, even physiological levels of calcium are adequate to non-proteolytically drive it from its zymogenic to activated conformation (heterotopic, the sequential and positive allosteric effect of sodium & calcium towards FXIII activation) (Fig. 6). Therefore, the extent and influence of calcium coordination on FXIII conformation also depends on the surface electrostatic state that in turn, is determined by the ionic strength/pH of FXIII´s solvent. The fact that ionic coordination alone does not influence protein conformation at the secondary structure level is further strengthened from our MD simulations in which we subjected only the core domain of the FXIII-A subunit to increasing concentrations of calcium (Fig. 4a). Again, as with high sodium concentrations, we observed a change in surface electrostatic potential with increasing calcium concentration (even though in the period of simulation, no actual coordination was observed for calcium as well). Therefore, the activity status of FXIII-A is not only the result of the coordination of calcium ions to its three binding sites; it is the net impact of calcium coordination as well the response to ancillary solvent ion concentration surrounding the molecule. Even physiologically FXIII-A exists in different solvent environments with differing ionic concentrations. Intracellularly FXIII-A is present as a dimer, and since no thrombin is accessible intracellularly, any enzymatic role, e.g., crosslinking of cytoskeletal proteins like actin/myosin is possible only through non-proteolytic activation^30–34^. Intracellular non-proteolytic FXIII activation could be achieved either through sudden ion fluxes like calcium release from endoplasmic reticulum that might alter the levels of calcium to supra-physiological levels fit for non -proteolytic activation^17^. The other option might involve an increase in sodium concentration, which, as mentioned earlier, will support non-proteolytic activation even at low concentrations of calcium. FXIII-A has been reported in various bodily fluids like placental fluid, tears, spinal fluid, etc. where it might have reparative roles^30,35–38^. If FXIII-A is considered as a therapeutic option for reparative processes related to these body fluids, knowledge of the subtle conformational changes brought about by the combination of ions in these fluids will help design easy to activate FXIII-A and with possibly a high specific activity.

### Energetic implications of calcium binding to FXIII explains why the activated form of FXIII-A is monomeric

**Figure 7:**
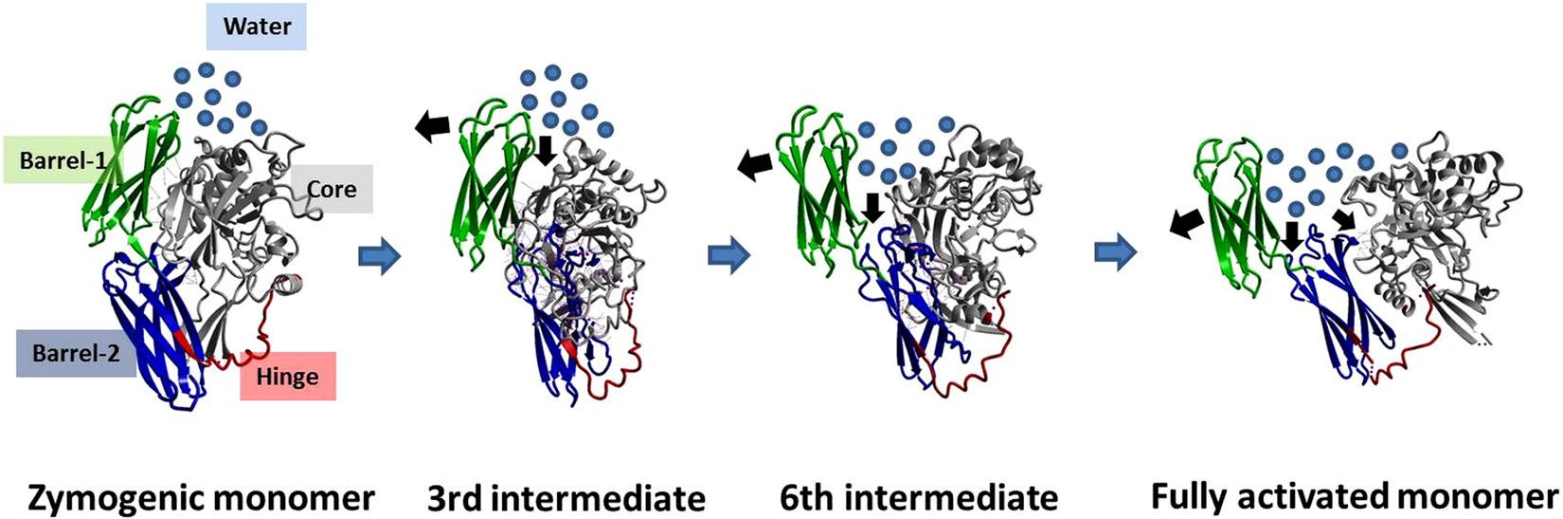
Entry of bulk solvent induces movement of β-barrel domains. Figure illustrates the stepwise conformational changes occurring during the activation of FXIII-A. The figure(s) represent in successive order the zymogenic crystal structure of FXIII-A monomer, the third and sixth transition state intermediate models reported earlier and the fully activated structure of FXIII-A monomer. All structures are depicted in the ribbon format. The N-terminal beta sandwich domain has been hidden for the sake of clarity. The β-barrel 1 and β-barrel 2 domains are coloured in green and blue respectively while the core domain is coloured grey. The hinge region of almost 20 amino acids is coloured red. The water solvation shell is depicted with blue circles. The movement of the β-barrel domains is depicted with black arrows. Contacts between the core and β-barrel domains are depicted with lightly coloured lines.

The FXIII complex assembly and its dissociation in plasma primarily involve the formation and disruption of non-covalent hydrophobic interactions^22^. Intrinsic ion binding to proteins involves hydration energies, which are entropically driven^39^. Also, calcium binding to transglutaminases is reported to induce conformational changes, as is observed in the crystal structures of activated forms of FXIII-A as well as TG2^15,22^.These conformational changes result in a change of hydrophobic surface area while exposing the core domain active site. Therefore, these changes are associated with an increase in entropy as is evident from our thermodynamic profile observed upon the sequential binding of calcium at c-value = 20 (entropy-driven, ∆H > 0 to ∆H < 0) (Fig. 5). This also is evidence of in-cell FXIII-A activation taking place upon calcium binding at these concentrations. Interestingly, the proteins belonging to the family bearing the ‘transglutaminase core’ including FXIII-A, are not part of the EF-hand superfamily (a superfamily of proteins bearing two EF-hand units, each is made up of two helices connected with calcium binding loop), which characteristically are calcium-binding proteins (SCOPe database; http://scop.berkeley.edu/). Calcium binding to proteins lacking an EF-hand motif involves the coordination of distal residues, made possible by changes in secondary structure/conformational changes as well as, major participation of a secondary ligand (like water) when required^40^. In our experiments with thrombin-cleaved rFXIII-A, the first step would be the saturation of Cab1. The ITC data suggest that the initial saturation event is highly spontaneous (∆G < 0), which is expected owing to the high affinity of FXIII-A towards calcium for Cab1 (100 µM)^3,17^. The high K_1_ for Cab1 would ideally be enthalpically favorable since this strong ion binding would constrict the molecular motions by bringing about an order in the spatial secondary structure^41–43^.This however is not the case with FXIII-A in which we observe a ∆H > 0/∆S < 0 patterns at Cab1 saturation indicating that there is a secondary event, most likely the coordination of Cab2, happening concurrently, that’s directed towards the transient disruption of Cab1 resulting in an overall absorption of energy which is reflected in the ∆H > 0/∆S < 0 patterns (Fig. 5d). Therefore, the first event cannot be independently thought of as binding of calcium at Cab1 but rather the combined saturation of Cab1 and simultaneous coordination of Cab2 to the point of transient disruption of Cab1. Once the Cab1 is transiently disrupted, the molecule overcomes the zymogenic constraint and moves conformationally towards the open, active structure as a result of a gain of entropy. This part of the activation cycle can be considered as the second binding event where we observe a pattern of ∆H < 0/∆S < 0. The gain in entropy (∆∆S) for this second event is higher than that observed for the first event, although effectively ∆G < 0, keeping the transformation to the activated form still favorable. At this point of time, the conformational changes taking place in FXIII-A have set into motion another event, which is the disruption of the zymogenic dimeric interface^16^. Major hydrophobic and non-covalent interactions between the opposing dimers are lost, resulting in (a) solvent-protected to a solvent-exposed state, and (b) exposure of Cab3. The third event in our thermodynamic cycle is the final coordination and saturation of Cab3, which is occurring simultaneously to the monomerization of the activated FXIII molecule. In this step, we observe a pattern of (∆H < 0, ∆S > 0) with the increase in entropy (∆∆S > 0), and release of heat (∆∆H«0) (Fig. 5d). This major increase itself represents a dissociative event combined with flexible inter-domain movements (of β-barrel domains). This event is aided by a major influx of water molecules in FXIII-A regions which were previously inaccessible to water (owing to the closed structural fold or the dimeric interface). The rise in entropy is the result of the enthalpic contribution of the final Cab3 saturation and dimer dissociation. This also keeps the overall free energy of the system negative (∆G < 0) favoring the final disruption of the dimeric interface to an open monomeric activated FXIII-A* form. The fast internal dynamics (conformational entropy, as a result of β-barrel domain movements), and slow internal dynamics (disruption of dimeric interface due to loss of hydrophobic interactions), leads to activation of a fully saturated FXIII-A* monomer, as the protein dissociates/solvate to a monomeric calcium saturated form (Fig. 7). One must, of course, remember that these events can occur in two different species, i.e., the dimeric FXIII-A_2_/intracellular FXIII-A_2_ and the plasma heterotetramer FXIII-A_2_B_2_ complex. In the latter, there are two dissociative processes in action, i.e., the dissociation of the FXIII-A_2_ zymogen to its activated monomeric FXIII-A* form and the dissociation of the FXIII-B_2_ molecule from the complex. Our thermodynamic experiments do not involve the FXIII-B subunit since they are performed with rFXIII-A_2_ alone. However, both dissociative events most likely run simultaneously each contributing to the success of the other. Both are brought about by conformational changes upon calcium binding and influx of water molecule into previous hydrophobic cores formed by the respective folds of the protein and the pattern in which the zymogenic complex is assembled.

**Figure 8:**
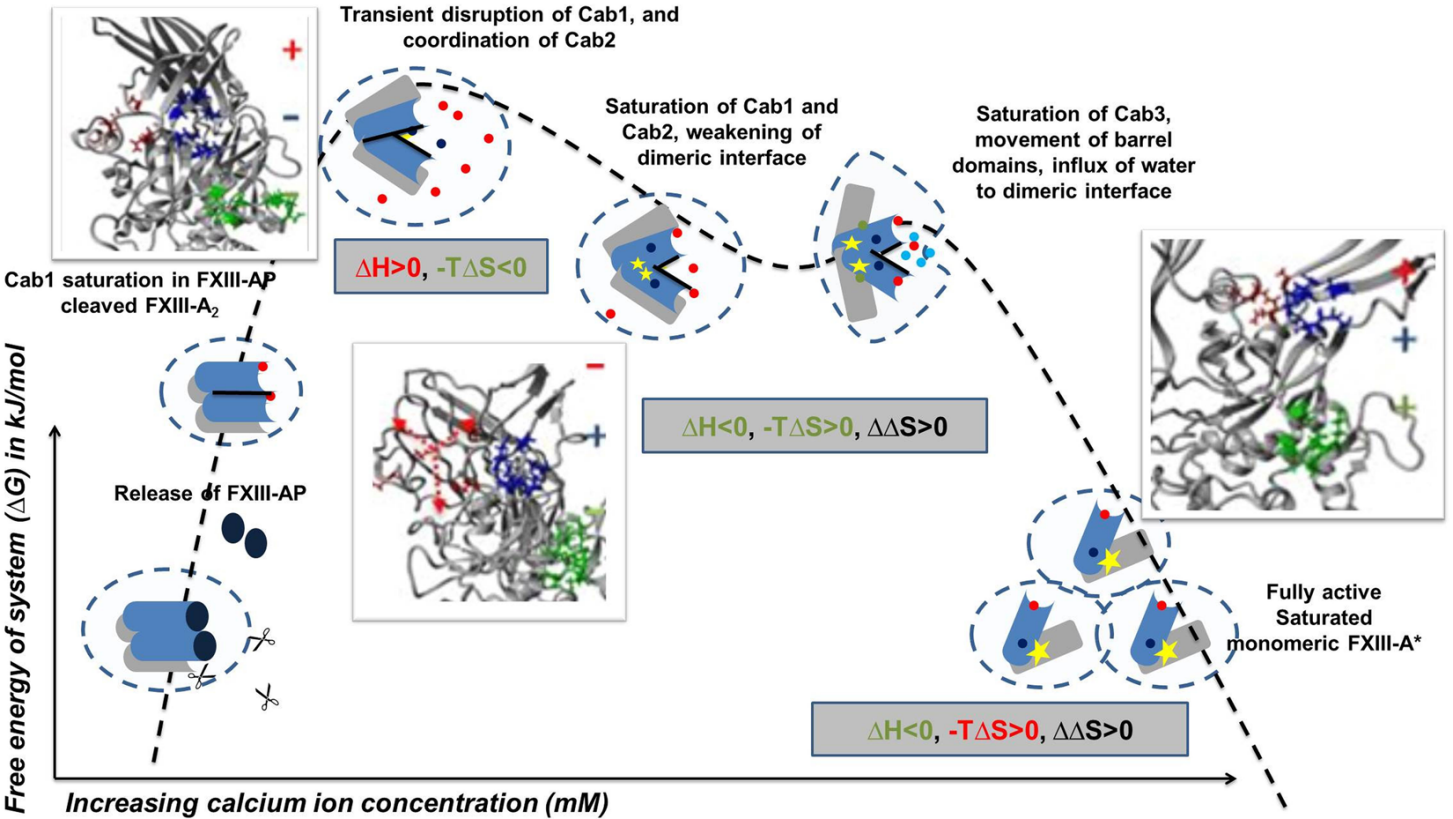
Derived Thermo-chemical Activation Cycle of Coagulation FXIII-A molecule in plasma. The free FXIII-A_2_ dimer, when devoid of any calcium possesses intact FXIII-AP and this species represents a low free energy stable form^16^. Cleavage of FXIII-AP by thrombin is followed by coordination and subsequent saturation of Cab1 and Cab2. Cab1 being spatially more accessible gets saturated faster and strongly than Cab2 yielding a low-energy, relatively stable state. Subsequently, the Cab2 coordination transiently disrupts Cab1 giving rise to a high-energy, unstable, transient state (event 1). The increasing saturation of Cab2 accompanied by the conformational changes directed by it result in the stabilization of unstable intermediate bringing down free energy. Meanwhile, slow disruption of the dimeric interface proceeds as the molecule proceeds towards full saturation (Cab3 gets exposed with the dimeric interface coming apart) (event 2). As the dimer loosens up, water molecules seep through and expose the dimeric interface further. Sharp movements of β-barrel domains as a consequence of calcium saturation, and movement of water molecules across the dimeric interface raise the entropy of the system, that finally culminate into dimer disruption, giving rise to monomeric, energy minimized, open active FXIII-A* species (event 3). Blue-grey object depict the N & C terminal of FXIII-A monomer, respectively. Molecule solvation shell is represented by dotted circle. Calcium ion saturation at Cab1, Cab2 and Cab3 is depicted by red, blue and green solid dots respectively. Black rod is the dimeric interface. Yellow star is the catalytic center. The three inset figure(s) demonstrate the secondary structure changes occurring during the course of activation explaining assembly (+) or disassembly (−) of calcium binding sites extracted from the Transition state intermediate models reported earlier^16^.

Studies on FXIII-A calcium binding explain the conformational changes occurring in the FXIII-A core domain, but how calcium binding contributes to the movement of the β-barrel domains was still unclear. A recent article shows that the first β-barrel domain protects the active site, and the second one is responsible for exposing it^42^. Our data highlights how enthalpy-entropy compensation contributes to the generation and stabilization of a monomeric FXIII-A* activated molecule upon calcium ion binding. Since the final event in the thermodynamic cycle of FXIII-A activation is enthalpically driven instead of entropically (i.e. like the first two events) this suggests that the first two events introduce disorder into the molecule (conformational changes) while the third and last event stabilizes the final molecule resulting from this disorder/conformational change, i.e., dissociation into a monomeric state. Our thermodynamic analysis highlights the role of the influx of bulk solvent that in continuation of the conformational changes induced by calcium binding enables the global domain movements observed during FXIII-A activation. At a structural level, we can observe these changes if we follow the conformation of the transition state models between the activated and zymogenic form of FXIII-A (Fig. 7). The β−1 and β-2 barrel domains in the zymogenic form are stabilized in through non-covalent bonds they form with the core domain that serves to shield the hydrophobic interior. The core domain is linearly linked to the β-barrel-1 domain by a hinge region which is unresolved in certain crystal structures of the zymogenic form like PDB ID: 1f13 because it is disordered and highly flexible. Therefore, barring the non-covalent attachment to the core domain, there is nothing preventing the β-barrel domains from moving flexibly over the hinge region. With the binding of calcium, conformational changes occurring at the core domain disrupt the non- covalent association of core domain with the barrel domains. This primary disruption enables the entry of the solvent molecules to this conformationally shielded region. Water influx further disrupts the β-barrel domain-core domain contacts, causing the β-barrel 2 domain to fall over under its weight, pulling the β-barrel 1 domain along with it in which the connecting hinge region acts as the pivot. This falling over motion appears as the twisting and opening up of the β-barrel domain to expose the catalytic site. We can, therefore, suggest that the formation of active FXIII-A* species involves a thermodynamic process of “conditional conformational switching”^29^, which involves the essential participation of calcium and water molecules. In other words, the activation of FXIII-A is majorly driven by conformational entropy brought about by calcium binding. Since all reactions follow a common energetic purpose, i.e. to favor the energetically stable complex/conformation, dimeric zymogenic FXIII-A has Cab2 and Cab3 sites hidden which make the dimeric, calcium unbound form more favorable in the zymogenic state (Fig. 8). During activation, since regions previously hidden get exposed, calcium binding alters conformation as well stabilize the final monomeric FXIII-A* molecule by bringing structural order upon coordination and reducing the transiently generated randomness. This explains why a monomeric bound FXIII-A* would be favored over the activated form (Fig. 8). It also explains why the chelation of calcium from a non-proteolytically activated FXIII-A, will shift the equilibrium to reversibly generate the zymogenic-dimeric FXIII-A that can be re-activated. Recent experimental evidence has also shown that the activated form of FXIII-A is, in fact, monomeric and is capable of reconversion to its zymogenic dimeric state upon removal of calcium from the medium^21^. Ever since FXIII-A has been recognized as a pharmaceutical candidate for inhibitor development, inhibitors have been designed primarily against the active site or the thrombin cleavage mechanism of this protein^44–48^. Our results suggest that two other areas of FXIII can potentially serve as regions against which inhibitors can be designed. The regions in and around the calcium binding sites belong to one group of potential candidates since they not only dictate the rate of activation but also the proper orientation of substrate binding sites. The other regions belong to the areas/cavities (next to β-barrel/core domain contacts) which allow the entry of bulk solvent upon the conformational changes induced by calcium binding. Neutralizing either of these candidate regions can potentially result in thermodynamically stabilizing intermediate conformational states of FXIII-A, thereby preventing the full activation of FXIII-A.

As a final commentary to this study, we would like to acknowledge some limitations of our study. Our work focusses on calcium binding without discriminating between proteolytic and non-proteolytic modes of activation^16^. A significant part of the work is based on transient transfections and *in silico* analysis performed on modeled structures. Similarly, the thermodynamic analyses are based on custom fitting models which themselves are built around hints from *in silico* experiments. We present this work to the readers as an exploratory characterization of individual calcium ion binding sites of FXIII-A. As future work, we will attempt to purify the mutants explored in this study to characterize them structurally/functionally in a pure, undiluted setting.

## Methods

### Transient heterologous expression of FXIII wild type and mutant variants

The human *HEK293T* cell line purchased from DMSZ (German Collection of Microorganisms and Cell Cultures) were cultured in high Glucose DMEM Invitrogen, supplemented with 10% v/v FBS (Invitrogen), 1% v/v Penicillin-Streptomycin antibiotics (Invitrogen) and 0.1% v/v Fungizone (Invitrogen), at 37 degrees in 5% CO2. All experiments were done on sub-cultured cells in logarithmic phase (below passage 20). Human FXIII-A cDNA, inserted into the cloning site of pDEST26^TM^ vector (Invitrogen) was used as a wild-type construct for all the experiments. Site-directed mutagenesis was performed on the aforementioned construct, using *GeneArt* Site-directed mutagenesis system (Life Technologies). Mutagenesis was performed with the aim of either accelerating calcium ion binding (*FXIII-A A264D, FXIII-A N347D, FXIII-A Q349D, FXIII-A A457D*), or disrupting calcium ion binding to the respective site (*FXIII-A N267K, FXIII-A D271K, FXIII-A D351K, FXIII-A E490K*) based on our previous hypothesis^16^. One more mutant at the thrombin cleavage site (*FXIII-A R38A*) was generated to be resistant to thrombin-mediated activation. Although a total of 13 different residues contribute to calcium binding in the three FXIII-A calcium binding sites, we were successful in cloning and expressing only 8 of them. All primers were synthesized by MWG Eurofins (Eurofins genomics). All vector construct clones were completely sequenced and verified for the correct incorporation of mutations *in-house*. Wild-type FXIII-A DNA and mutated DNA were transfected into mammalian *HEK293T* cells for transient expression. Briefly, 2.7 × 10^5^ cells were transfected with 3 µg of plasmid DNA along with 6 µl of transfection reagent lipofectamine 2000 (Invitrogen). Cultures were harvested 48 hours post-transfection, by performing intracellular lysis using mammalian native M-PER reagent (Thermo Fischer Scientific Inc), containing 25 mM bicine, pH 7.6, supplemented with 0.1 mM PMSF for 10 minutes and centrifuged at 14,000 g for 5 minutes at 4 °C. Lysates were stored at −70 °C for later evaluation. Each transfection lot was accompanied with a positive control, i.e., *HEK293T* cells transfected with expression vector expressing eGFP. This lot helped us evaluate the success of the transfection as well as check for any endogenous FXIII/transglutaminase activity. Results for all assays performed on the transfection lysates were normalized accordingly.

### Antigenic estimation of rFXIII-A by FXIII-A Western Blot Analyses

Intracellular lysate derived from transiently transfected *HEK293T* cells was verified for the antigenic presence of FXIII-A protein by western blot analysis. Intracellular lysate from transfected cells was quantified by BCA estimation (Pierce, Life technologies). The intracellular lysate was separated on 4–16% precast SDS PAGE (Biorad), followed by transfer to PVDF membrane at 60 V for 90 minutes in cold room. The membrane was blocked for 1 hour at room temperature in blocking reagent (3% w/v BSA in PBS with 0.05% Tween-20). Subsequently, after a wash with PBS-Tween (0.05%), the membrane was incubated for 1 h at room temperature in Primary antibody (1 µg/mL) (Rabbit-anti human Factor XIII-A polyclonal antibody, Thermo Fischer Scientific Inc) with mild shaking. After washing thrice in PBS-Tween (0.05%), the membrane was incubated for 1 h at room temperature in HRP tagged Secondary antibody (50 ng/mL) (Anti-Rabbit HRP, Thermo Fischer Scientific Inc). Finally, the membrane was washed thrice in PBS-Tween and PBS, respectively. Chemiluminescent signal quantification, Image acquisition (ChemiDoc MP, Bio-Rad) and densitometric evaluation of signal were performed on Image lab Software (Bio-Rad) version 4.1. Using known amounts of recombinant FXIII-A (positive control), the percentage antigenicity and absolute quantity of antigenically active FXIII-A was calculated in transfected samples based on the chemiluminescent intensity of the signal. The antigenic levels determined by densitometry were confirmed with a commercial sandwich ELISA (AssayMax). The antigenic levels were used in combination with activity levels from photometric assay to calculate the specific activity of the individual samples.

### FXIII activity determination by biochemical end-point assays

#### Photometric assay

Intracellular lysates (Wild type; Mutants), were 6X concentrated using Amicon filters (30 kDa cut-off) (Merck Millipore) for the evaluation of FXIII activity with a kinetic photometric assay (Berichrom, Siemens, Germany). Briefly, samples were activated in the presence of thrombin and calcium, at 37 °C, transglutaminase activity of FXIII-A was measured indirectly by measuring released indicator, i.e., Ammonia (Abs 340 nm). A decrease in absorbance is directly proportional to FXIII-A transglutaminase activity in the samples^49^. The assay was performed on the Behring Coagulation System® (BCS) (Dade Behring, Marburg, Germany). All the experiments were performed in duplicates, with three sets of transfections to ensure reproducibility.

#### Pentylamine incorporation assay

The activity of intracellular lysates of recombinant factor XIII (rFXIII) Wild type, Mutants and a negative control was determined based on a pentylamine incorporation assay as described previously^50^. Briefly, microtiter plates were coated with 80 µg/mL human fibrinogen (Enzyme Research Laboratories, UK) at 37 °C for 1 hour, then blocked with 1% BSA overnight at 4 °C. Plates were incubated with duplicates of 10 μL lysate, 0.27 μM 5-(Biotinamido) pentylamine (Thermo Fisher Scientific Inc), 1 U/mL human thrombin (Calbiochem, Merck KGaA), 100 μM Dithiothreitol (Sigma), and 1 mM CaCl_2_. Incorporation of 5-(Biotinamido) pentylamine was stopped with 133 mM EDTA after 0 or 30 minutes. Cross-linking of the 5-(biotinamido) pentylamine into the fibrin by recombinant FXIII was detected using streptavidin-alkaline phosphatase (Life Technologies) and p-Nitrophenyl phosphate (Sigma). Plates were measured at OD 405 nm in a Powerwave Bio-Tek multiwell plate reader (Winooski, USA). Optical density (OD) values at time 0 were subtracted from the 30-minute readings for each lysate to remove background and a standard curve of known concentrations of FXIII were used to extrapolate activity of rFXIII in each lysate.

#### Determination of protein activity by α2-antiplasmin incorporation

The activity of intracellular lysates containing recombinant factor XIII (rFXIII) wild type, mutants, and a negative control was also assayed by α_2_-antiplasmin incorporation, based on a method previously described^51^. Briefly, microtiter plates were coated with 80 µg/mL human fibrinogen (Enzyme Research Laboratories, UK) at 37 °C for 1 hour, then blocked with 1% BSA overnight at 4 °C. Plates were then treated in duplicate with 10 μL lysate, 10 µg/mL α_2_-antiplasmin (Calbiochem), 1 U/mL human thrombin, 0.1 mM DTT, and 10 mM CaCl_2_. Incorporation of α_2_-antiplasmin was stopped with 133 mM EDTA after 0 or 60 minutes. Cross-linking of the α_2_-antiplasmin into the fibrin by rFXIII was detected using goat anti-human α_2_-antiplasmin antibody with a horse-radish peroxidase conjugate (Enzyme Research Laboratories) and 1, 2-diaminobenzene *o*-phenylenediamine (OPD; Dako). Plates were measured at 490 nm in a multi-well plate reader (Bio-Tek). OD values at time 0 were subtracted from the 60-minute readings for each lysate to remove background and a standard curve of known concentrations of FXIII were used to extrapolate activity of rFXIII in each lysate.

### FXIII activity determination by a continuous FXIII-A* generation assay

Activated FXIII-A (FXIII-A*) generation was triggered by tissue factor/phospholipids (TF/PL), and FXIII-A* isopeptidase activity was measured using the fluorogenic substrate A101 (Zedira, Darmstadt, Germany) in a Safire microtiter plate reader (Tecan, Crailsheim, Germany)^26^. Twenty-five microliters FXIII-deficient plasma (deficient for FXIII-A_2_ and FXIII-B_2_; Haemochrom Diagnostica GmbH, Essen, Germany) spiked rFXIII-A_2_ mutants (equal amount of crude samples based on antigenicity) were incubated with 35 μL reagent solution (5 μL 100 mM glycine methyl ester, 5 μL 2 mM fluorogenic FXIII-A* substrate, 10 μL Innovin (recombinant TF; Dade Behring, IL, USA) diluted 1:700 in phospholipids (PTT reagent kit, Roche,USA) and 15 μL HBS (20 mM Hepes, 150 mM NaCl)/0.1% serum albumin pH 7.5. The reaction was started with 40 μL HBS pH 7.5 containing 25 mM CaCl_2_. For the thrombin resistant mutant FXIII-A-R38A, increasing amounts of calcium (25 mM, 50 mM, 100 mM, and 200 mM) in HBS pH 7.5 were used. Fluorescence was measured over 1 hour at excitation wavelength = 330 nm and emission wavelength = 430 nm in kinetic mode with data acquisition 2 times per minute. All the experiments were performed in duplicates, with three sets of transfections to ensure reproducibility. Human standard plasma (Siemens Healthcare, Erlangen, Germany) was used as an internal assay control.

Three parameters (further explained in the statistical analysis section) were obtained for each variant and the wild type from the FXIII-A* continuous generation curve:

(A) Rate of FXIII-A* generation/maximal rate (µ; expressed as ∆R.F.U/min) which represents the rate of conformational change between the zymogenic and activated forms. The transglutaminase crosslinking reaction is a two-step reaction (a ping-pong mechanism), in which two substrates sequentially accesses the active site to first form intermediates and then get crosslinked to each other^22,25^. Since the continuous generation assay relies on the isopeptidase activity which involves a singular cleavage/hydrolysis of a quencher attached to the substrate peptide immediately post the formation of the activated FXIII-A*, it is more reflective of the change in conformation between the zymogenic and activated FXIII-A forms than any end-point assays. Theoretically, both continuous-generation assay and end-point activity assay should show correlation, but that might not be the case for mutant variants, especially those that alter substrate binding sites.
(B) The tlag is the time required for the activation of FXIII-A by thrombin (in plasma background), in the generation assay. Since in generation assay, the activation process is initiated by Innovin, a recombinant tissue factor and which is upstream to the thrombin cleavage of FXIII-A in the coagulation pathway, there is always some time lag before the first signal of activated FXIII-A* can be recorded. Any delay or faster tlag might signify differences in thrombin cleavage of FXIII-A.
(C) The tmax is the time taken to reach the maximal rate µ.

(Note: An illustration of the generation assay curve along with a small commentary is provided in Supplementary Fig. 2 to help the reader understand the assay better).

### Mapping substrate binding sites on FXIII-A subunit activation transition state intermediate models

We used FXIII-A subunit activation transition state intermediate models we had earlier generated and reported, to study the changes in the substrate binding regions as the FXIII-A molecule unfolds from its closed zymogenic state to its open fully activated state during the process of activation^16^.Three major putative substrate binding regions i.e. fibrinogen, BAPA and α-2 antiplasmin were considered since the endpoint FXIII activity assays dealt with these substrates. These binding regions had been determined by rigid docking studies we had earlier conducted and reported for fibrinogen and α-2 antiplasmin^52^.The putative BAPA binding region(s) were predicted by docking the structural coordinates of BAPA [5-(Biotinamido) pentylamine; Pubchem ID: CID 83906] that were downloaded from Pubchem database (as an SDF file and later converted to a PDB file on YASARA), onto the activated FXIII-A crystal structure (PDB ID: 4kty) downloaded from the protein database. Semi-flexible docking was performed with the Autodock function embedded in YASARA^52^.Finally these binding regions were mapped and highlighted on the FXIII-A subunit activation transition state intermediate models.

### Molecular dynamic simulation of FXIII-A at different ionic concentrations

The effect of increasing ion concentration on the structure of FXIII-A subunit was studied by running plain Molecular dynamics (MD) simulation the zymogenic human FXIII-A_2_ crystal structure (PDB ID: 1f13; 2.1 Å resolution) and only the core domain of FXIII-A subunit (isolated from the PDB ID: 1f13 and consisting of the amino acids between 183–515 residues) on the YASARA Structure package version 13.11.1 platform^53,54^. Gaps or unresolved regions within the crystal structure(s) were modelled on the FREAD loop modelling server (http://opig.stats.ox.ac.uk/webapps/fread/)^55^, e.g., the PDB file 1f13 that has missing regions at the thrombin cleavage site Arg37-Gly38 was submitted to the server under default parameters and with the starting and ending residue of the missing region specified. The final gap resolved structure was chosen from the output file based on scores that were a combination of all backbone atom anchor match RMSD (corresponds to the base structure) and all backbone atom loop match RMSD (corresponds to the loop structure). The PDB files were initially subjected to a 500 ps refinement MD simulation run that imposes the YAMBER3 force field parameters in YASARA in order to remove steric clashes and improve rotamer geometry^53^. The file with the lowest energy in the simulation trajectory was chosen for conducting further simulations. Simulations were performed with the md_sim macro embedded in YASARA. The macro was modified for running simulations at different ionic concentrations. Briefly, a simulation cell with periodic boundaries and 20 Å minimum distances to protein atoms was employed with explicit solvent. The AMBER03 force field, NPT ensemble was used with long range PME potential and a cut-off of 7.86Å^56^. Hydrogen bond networks were optimized using the method of Hooft and co-workers^57^. The simulation cell was filled with water at a density of 0.997 g/mL and a maximum sum of all bumps per water of 1.0 Å. Most importantly, the simulation cell net charge was neutralized with different NaCl and CaCl_2_ concentrations. While the full zymogenic dimeric structure was simulated at 150 mM and 1 M NaCl, the core domain was simulated at 5 mM, 10 mM, 50 mM, and 100 mM CaCl_2_. The entire system was energy minimized by steepest descent to remove conformation stress within the structure, followed by simulated annealing minimization until convergence was achieved. The MD simulation was performed at 298 K. Simulations for all structures at all concentrations were run for a minimum of 100 ns after equilibration was achieved. Secondary structure content during the MD simulations was visualized using the md_analyzesecstr macro output embedded in YASARA on R. Structural image visualization, analysis, and rendering were done with YASARA 13.11.1 and Chimera version 1.10.2^54,58^. Electrostatic surface potential was calculated and graphically depicted using the Adaptive Poisson-Boltzmann Solver integrated within YASARA^59^. The inter-residue interaction or Residue interaction network (RIN) within a structure was visualized by submitting the PDB file corresponding to that structure (usually the 100 ns simulation snapshot for all simulated structures in this study) to the RING 2.0 server (http://protein.bio.unipd.it/ring/)^60^. The server identifies covalent and non-covalent bonds in protein structures, including π–π stacking and π–cation interactions using a complex empirical re-parameterization of distance thresholds performed on the entire submitted PDB file. The output is in the form of a colour coded point and connector network pattern. The colour codes are explained in the inset diagram for all network pattern outputs.

### Conservation of calcium binding sites within FXIII-A subunit

The conservation of the calcium binding site residues within the FXIII-A subunit were analyzed on the *ConSurf* server (http://consurf.tau.ac.il/2016/)^61^ which is a bioinformatics tool for estimating the evolutionary conservation of amino/nucleic acid positions in a protein/DNA/RNA molecule based on the phylogenetic relations between homologous sequences^61^. The degree to which an amino (or nucleic) acid position is evolutionarily conserved (i.e., its evolutionary rate) is strongly dependent on its structural and functional importance. The FXIII-A sequence from UniProt (ID: P00488) was submitted to this server under default conditions (Homolog search algorithm: HMMER; E value cut off at 0.0001; Proteins database: UNIREF-90; Alignment method: MAFFT-L-INS-i; Calculation method: Bayesian; Evolutionary substitution model: Best model) but with higher number (n = 5) iterations. The resulting alignment output was viewed on the Jalview alignment viewer. The structural conservation of the calcium binding sites was evaluated by structurally aligning all human transglutaminase structures, including FXIII-A subunit using the Multiseq tool embedded in VMD^62^. All biophysical structures for FXIII-A subunit (PDB ID: 1f13), TG2 (PDB ID: 4pyg) and TG3 (PDB ID: 1nuf) that are currently resolved and available were downloaded from the protein database for this purpose^15,29,63^. The remaining transglutaminases with no known structures were modeled on *I-TASSER* modeling and threading server^64^. The sequences of these transglutaminases (extracted from the Uniprot database) were submitted to the *I-TASSER* server under default conditions. The highest scoring (best C-score) model among the output files was chosen for multiple alignments (Supplementary Fig. 9a–d). Post alignment, a structure-based phylogenetic tree was generated using delta QH values, which is a measure of structural homology. Subjects closer to each other on this tree are structurally similar.

### Thermodynamic analyses of Calcium binding to FXIII-A by Isothermal titration calorimetry

Isothermal titration calorimetry experiments were carried out on a MicroCal200 microcalorimeter (Malvern Panalytical, UK). The reference cell was filled with Autoclaved MiliQ water. The rFXIII-A_2_ (expressed in-house in Yeast expression system) in the sample cell was titrated against calcium concentrations. The sample (rFXIII-A_2_) was re-suspended in 20 mM Tris pH 8.2 before titration. In principle, the titrations were performed based on the c-value (concentration of calcium in syringe/concentration of sample rFXIII-A_2_ in the cell). Since plasma FXIII and calcium ion concentration are 2 mg/L (an effective molar concentration of 0.0125 µM) and 2.5 mM respectively, the c-value for mimicking a plasmatic environment will be almost 200,000 which is beyond the sensitivity of ITC^65–67^. Hence, titrations were performed at a high c-value as 25,000; and a standard c-value of 20^67^. For each of the conditions, titrations were performed in the same buffer (20 mM Tris, pH 8.2), unless stated otherwise. For c-value = 20, 1.25 mM of rFXIII-A_2_ was titrated against 25 mM CaCl_2_ (with 13.8U thrombin (Sigma) in the sample cell). The titration involved 19 injections (12 × 2, 0.4 × 6, each injection spaced by 120 s). To ensure complete saturation, the reaction was continued with a further 19 injections (12 × 2, 0.4 × 6, each injection spaced by 120 s). Both the resulting isotherms were concatenated with CONCAT tool provided with the Origin software (version 7.0) (OriginLab)^68^. For c-value = 25,000; 1 × 10^−3^ mM rFXIII-A_2_, activated by 2U of thrombin (Sigma) was titrated against 25 mM calcium. The titration was performed for 19 injections 2 µL each, spaced at 150 s, to reach full saturation, the reaction proceeded with further 19 injections (0.4 µL each, spaced at 150 s) and the titrations were concatenated with CONCAT tool provided with the Origin software (version 7.0). All the experiments were performed in the same buffer (20 mM Tris, pH 8.2), at 30 degrees with the stirring speed set to 750 rpm, at low feedback. For each experiment, thrombin was added before the start of the reaction; hence rFXIII-A_2_ was incubated with thrombin, in the absence of calcium for the period of pre-titration delay. To account for the heat of dilution, we performed blank experiments under the same conditions without rFXIII-A_2_ in the sample cell (Thrombin in cell titrated against CaCl_2_ in the syringe). Peak integration was done in the software Origin 7.0. For thermodynamic analysis, initially, we used Origin software, for single set of binding model, in order to observe the binding as a global fit. The parameter K_d_ was approximated around 100 µM based on earlier reports, was set as non-varying parameter for sequential binding site model with 3 set of binding sites (Even for sequential binding site model, it is advisable to have initial guesses for n, K and ∆H)^3,69^. (The K_1_ was used as a standalone non-varying parameter for the further fitting of data in all fitting exercises). Subsequently, heat capacity changes for each injection were calculated based on the algorithms followed by Origin software, as well as stoichiometric equilibria model (described below) in *Affinimeter* (https://www.affinimeter.com)^70^ and the process was iterated until no further significant improvement in fit were observed. Individual heat-signatures were derived from the energy definitions upon each binding after model fitting also by using *Affinimeter*. The corresponding thermodynamic parameters were calculated according to the equation ln(1/K_d_) = (∆H-T∆S)/RT. Additionally, titrations were performed at 1 µM and 0.4 µM FXIII-A_2_, with ligand (calcium ion) concentration of 12 µM and 14 µM respectively (for c-values 12 and 35) (data not shown), and no binding was observed in these titrations. The titrations were first simulated in *Affinimeter* with the aforementioned c-values & fitting was simulated to the following custom design model, using the model-builder approach on *Affinimeter* app to dissect the contribution of individual species generated during the course of FXIII-A activation, following the hypothetical equation:

$${M}1+{A}1\leftrightharpoons {M}1{A}1+{A}1\leftrightharpoons {M}1{\boldsymbol{A}}2+{A}1\leftrightharpoons {M}1{A}3$$

where M is FXIII-A_2_, A is calcium ion.

The final data was interpret based on fitting exercises performed using *Affinimeter*.

### Statistical analysis

Statistical analysis was performed using R^69^. For endpoint assays, mean values were compared (to the wild type) by Student’s two-tailed t-test. In the generation assays, data was analyzed based on growth-curve analyses, with the slope of the curve (µ) representing the growth rate. Data were fit using R-package *“Grofit”*, based on the dose-response relationship^71^. Non-parametric spline estimation was done to fit the data, and to obtain the characteristic parameters lag phase (tlag), maximal growth rate (µ), time of maximal growth rate (tmax), maximal growth (A) and area under the growth for each single growth curve.

The function follows the following parameterization***:***

$$y(t)={A}^{\ast }\,\exp [\,-\,\exp ({\mu }^{\ast }\,\exp (1)/{{\rm{A}}}^{\ast }\,(\lambda -t)+1)]$$

Abbreviations: lag phase (tlag): λ maximal growth rate (rate of activation): **µ**, Area under curve: **A**, and time of observation: **t**

## Supplementary information

Supplementary file

## Data Availability

The datasets generated during and/or analyzed during the current study are available from the corresponding author on reasonable request.
