# Supplementary material for: Structure functional insights into calcium binding during the activation of coagulation factor XIII A
Source: Sci Rep. 2019 Aug 5;9:11324. doi: 10.1038/s41598-019-47815-z (PMC6683118; doi:10.1038/s41598-019-47815-z)
Supplement: Supplementary file 1 — Supplementary file [file 41598_2019_47815_MOESM1_ESM.docx]

**Supplementary Material**

**Structure functional insights into calcium binding during the activation of coagulation factor XIII-A**

Sneha Singh, ^a^ Johannes Dodt,^b^ Peter Volkers,^b^ Emma Hethershaw,^c^ Helen Philippou,^c^ Vytautus Ivaskevicius,^a^ Diana Imhof,^d^ Johannes Oldenburg,^a^ and Arijit Biswas^a^^[[1]](#footnote-1)^*

*^a^ Institute of Experimental Hematology and Transfusion medicine, University Hospital of Bonn. Bonn 53127 Germany*

*^b^ Paul-Ehlrich Institute, Langen 63225 Germany*

*^c^ Discovery and Translational Science Department, University of Leeds, Leeds LS29JT, United Kingdom*

*^d^ Pharmaceutical Biochemistry and Bioanalytics, Pharmaceutical Institute, University of Bonn, An der Immenburg 4, D-53121 Bonn German)*

**Supplementary Figure 1:** The high-scoring docking poses of BAPA on the activated FXIII-A crystal structure (PDB ID: 4kty).


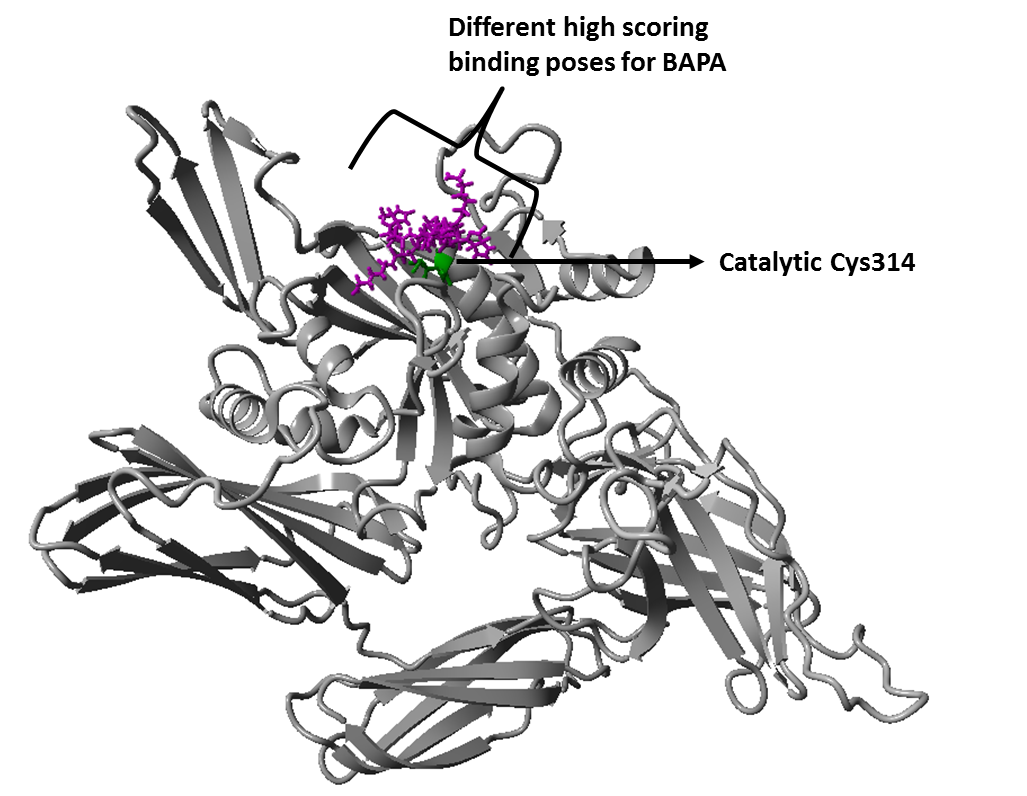


**Supplementary Figure 2.**

**
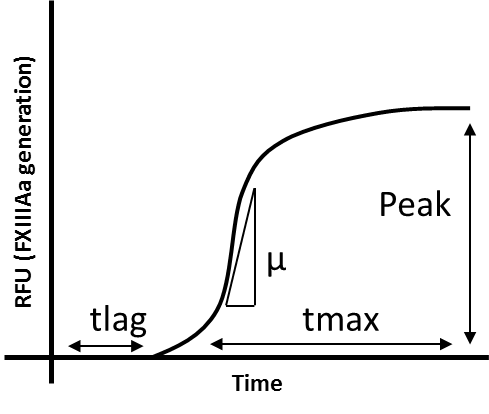
**

**Additional Commentary on FXIII-A* Generation assay**

The FXIII-A* Generation assay, was introduced by Dodt et al, in 2013^2^ introduces a new kinetic, real-time, quantitative approach to determine the rate of active FXIII-A* molecule generation and also its depletion in a given amount of time. This method is majorly an extension of flourimetric assay versus the coupled photometric assay used in routine widely. Based on the “iso-peptidase” activity of FXIII-A molecule, this in-vitro assay mimcs close resemblance to the in-vivo plasma based matrix (performed in FXIII-deficient plasma), employing the combination of thrombin generation assay and flourogenic assay, detecting the conversion of a flourogenic substrate to product as a result of isopeptidase activity of FXIII-A molecule. Theoretically, FXIIIa substrate (A101 from Zedira GmBh, Germany) will occupy free FXIIIa, and the ideal substrate would have a high Km, leaving enough FXIIIa for adequate reactions with other plasma substrates or inhibitors. Substrate A101 binds to free FXIII-A* and falls off, binding gives fluorescence µ represents available free active FXIII-A* molecules. The curve data was evaluated according to a bi-exponential model with first order absorption and elimination. Data were fitted to the equation:


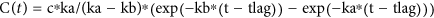


where ka – constant of absorption which describes the development of FXIII-A* and kb – elimination constant. The parameters area under the curve (AUC), peak FXIII-A* concentration (CP), and time to peak (TTP) were also evaluated.

The growth function follows the following parameterization for data analyses^3^***:***

***y(t)=A*exp[-exp(µ*exp(1)/A*( λ-t)+1)]***

*Abbreviations: lag phase (tlag): λ maximal growth rate (rate of activation): µ, Area under curve: A, and Maximal time to peak (tmax): t*

**Supplementary Figure 3:** Generation assay curves obtained for the mutant FXIIIA-R38A at increasing concentrations of calcium. The trendline depicts the effect on rate of generation upon increasing calcium ion concentration. The figure shows the values obtained from the fit with R squared value.


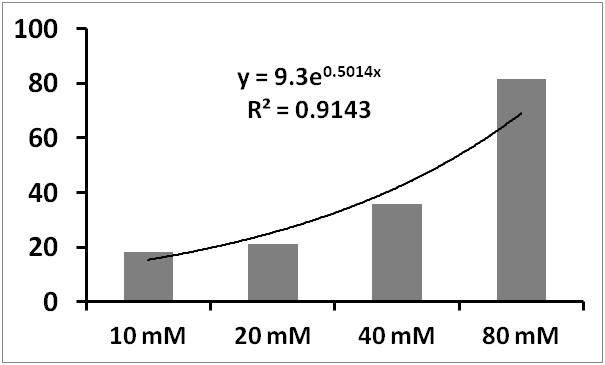


**Supplementary Figure 4:** The post-equilibration RMSD graphs (in Å), for simulations performed at different sodium and calcium concentrations on the full length FXIII-A crystal structure (upper two panels) and its core-domain (lower four panels) respectively. The X-axis represents increasing simulation time (in ns), for each of the graphs.


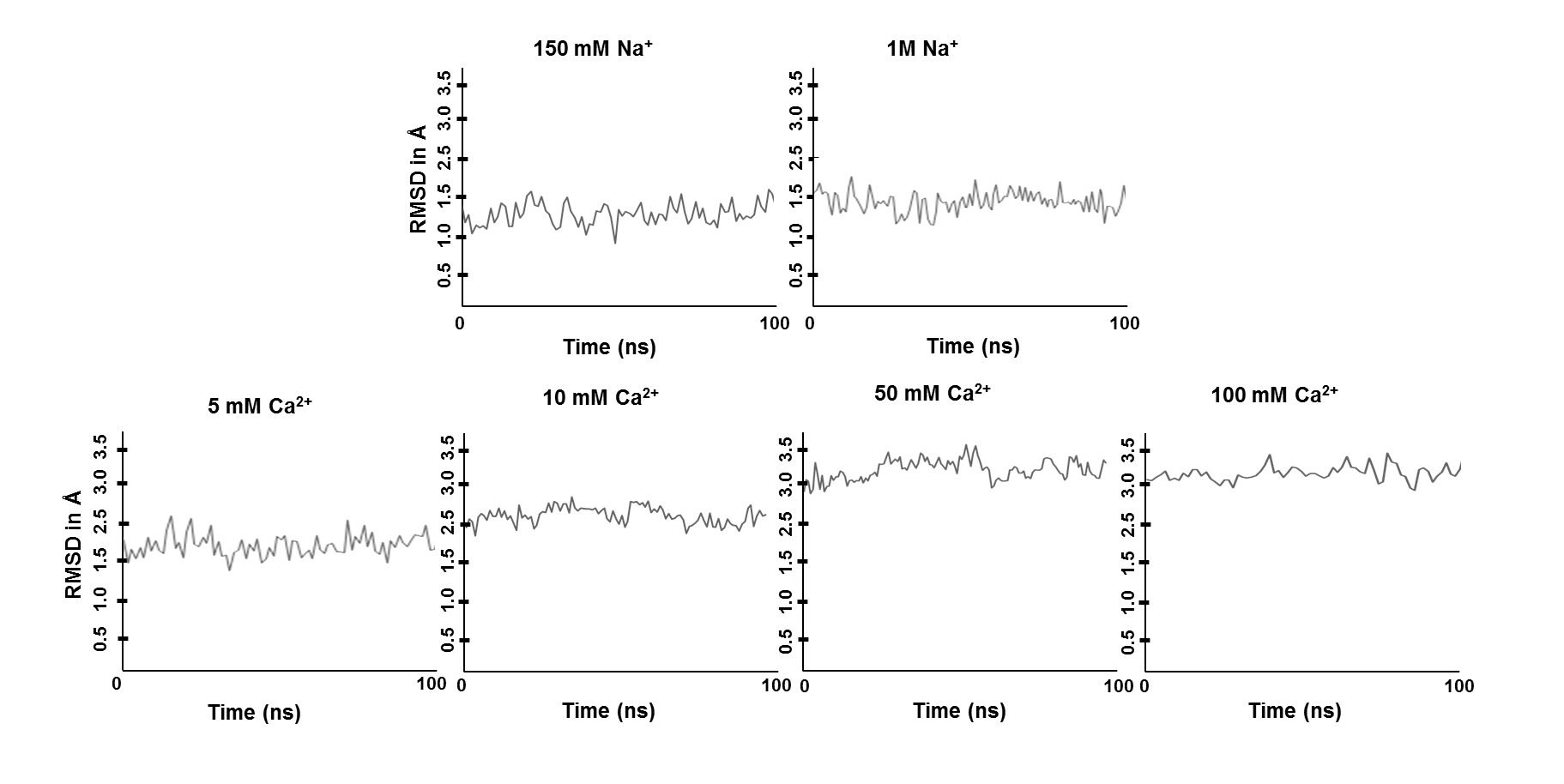


**Supplementary Figure 5: Fitted isotherms for ITC titration of rFXIII-A (protein in sample cell) vs CaCl_2_ (ligand in syringe**), as calculated in Affinimeter following the stiochiometric binding equilibria explained in main Figure 5A. **Left:** Titration performed with c-value 25,000 (rFXIII-A_2_ is 1 µM in cell and Ca^2+^ is 25 mM in syringe). The protein seems to undergo oversaturation at first few injection of titration (depicted by squared peaks in the raw data (supplementary figure 6b)). **Right:** Titration performed with c-value 20 (rFXIII-A_2_ is 1.25 mM in cell and Ca^2+^ is 25 mM in syringe). On following the stoichiometric binding equilibria following the model (in main Figure 5A), the data fits with a chi-squared value of 1.31E^1^. (Exp is experimental value, Fit is fitted value, and delta is the difference).


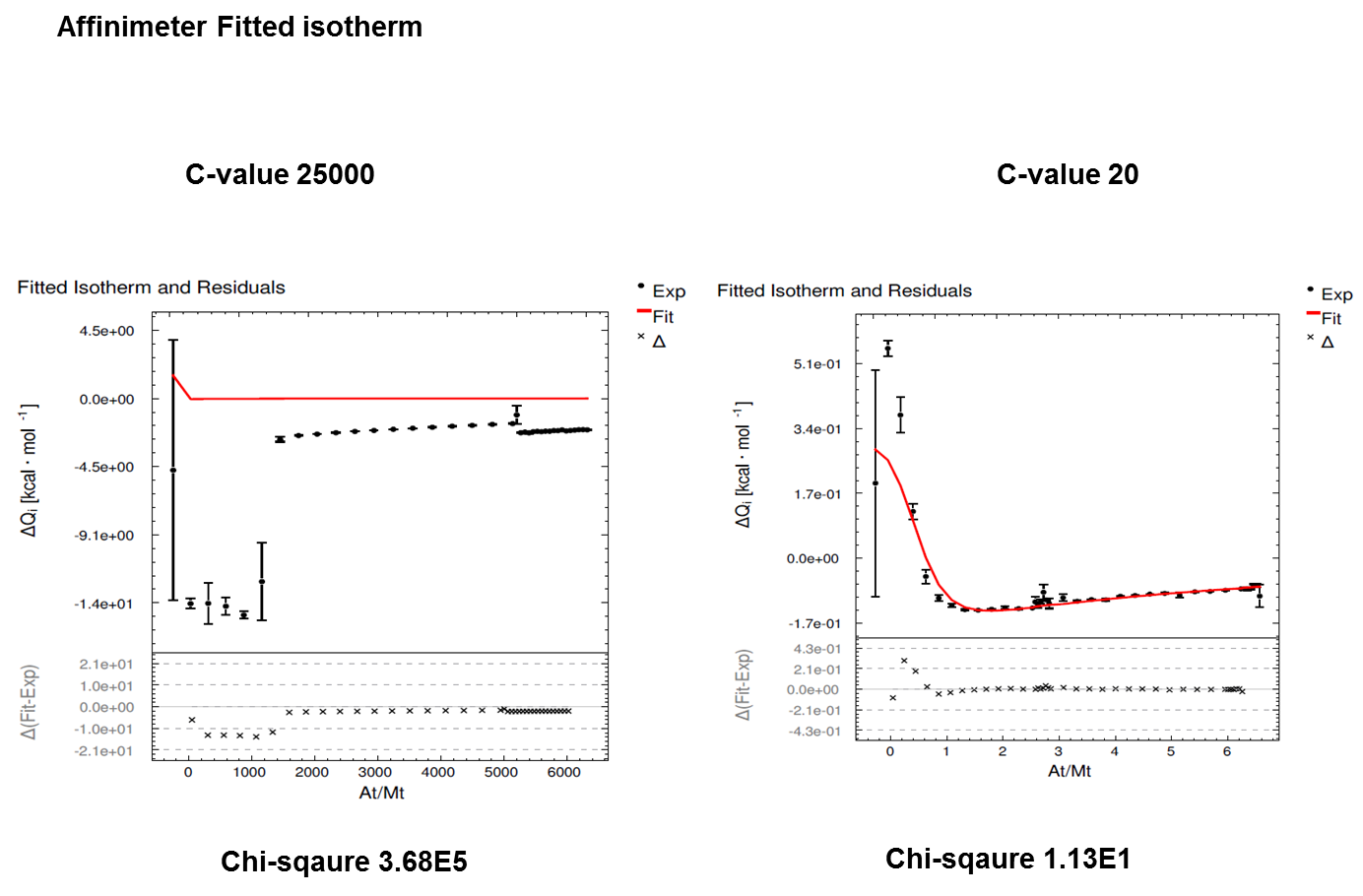


**Supplementary Figure 6:** **Calcium binding to rFXIII-A_2_ studied by Isothermal titration Calorimetry.** **Panel** **a**. Equation depicting the stoichiometric binding equilibrium model, followed for the analysis of data derived for ITC (Model was generated in Affinimeter using model builder approach). **Panel b**. Titration of 1 µM rFXIII-A_2,_ with 25 mM CaCl_2_ (**c-value=25,000**) Upper figure in this panel is the raw data depicting the heat change upon each injection, lower image is the normalized data, with integrated heat change plotted against the concentration ratio of calcium vs rFXIII-A_2._ Solid black line represents the corresponding fit obtained in Origin software using sequential binding mode, with n=3. All heat changes are plotted after subtraction of reference (no rFXIII-A2 in sample cell vs. 25mM CaCl_2_ in the presence of Thrombin, at same conditions). **Panel’s c, d and e** are based on evaluation performed on Affinimeter. **Panel c**, depicts the contribution of individual species, generated during a sequential binding of Calcium to FXIII-A (based on earlier observations (19) and as participating of the Stiochiometric equilibria here (Panel A)), towards the binding isotherm. **Panel d** is the heat signature, or the thermal footprints obtained at each binding event following the equation ∆G = ∆H - T∆S (2nd Law of Thermodynamics). **Panel e.** is table representing event-wise changes in enthalpy (∆H), in kJ/mol, and corresponding change in binding affinity of calcium ions towards FXIII-A.


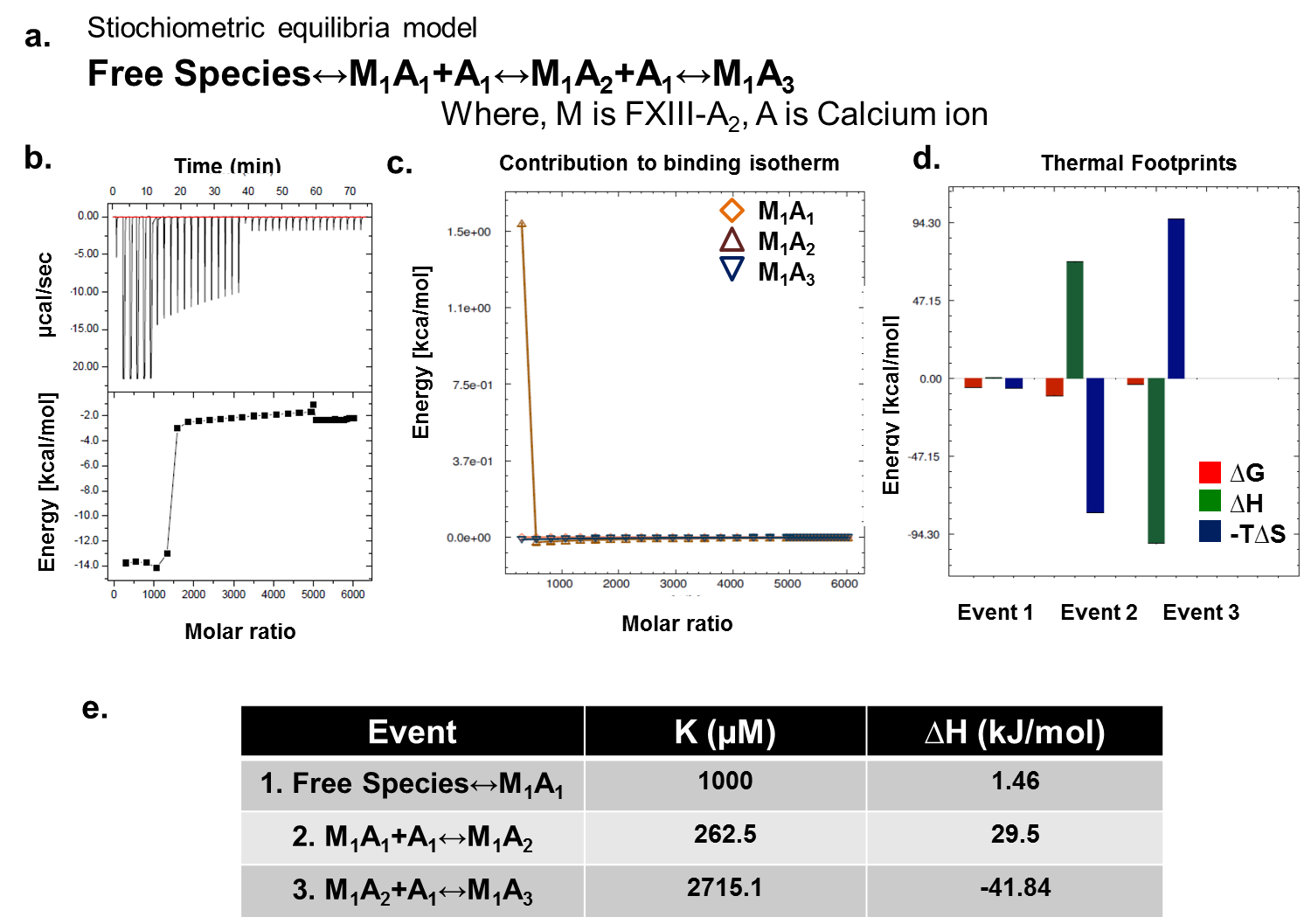


**Supplementary Figure 7:** Spatial alignment of the three calcium binding site residues of FXIII-A with TG1 (Panel A) and TG7 (Panel B). The calcium binding sites are named and numbered based on the calcium binding sites reported on FXIII-A.


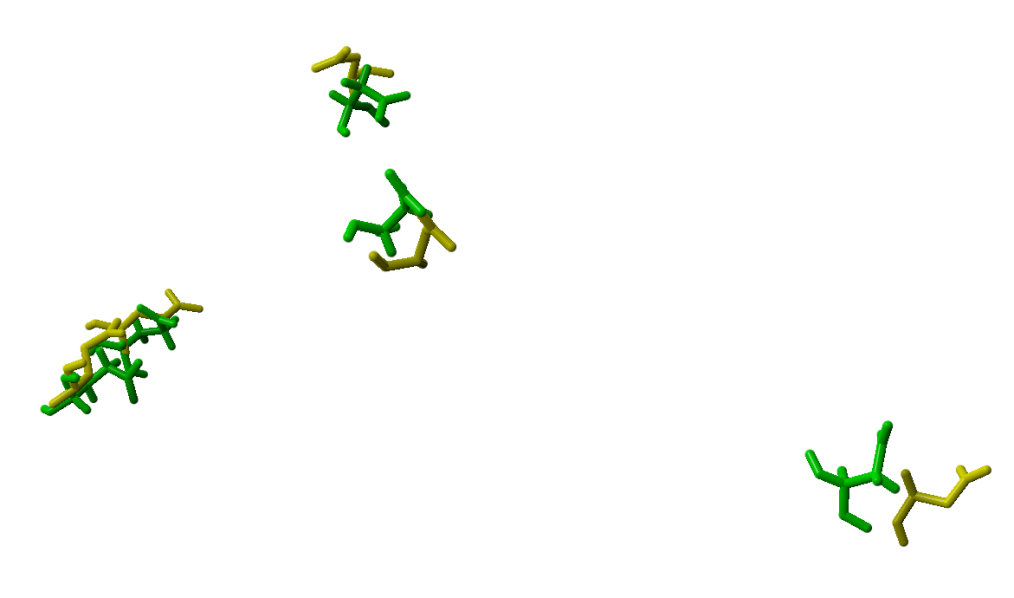

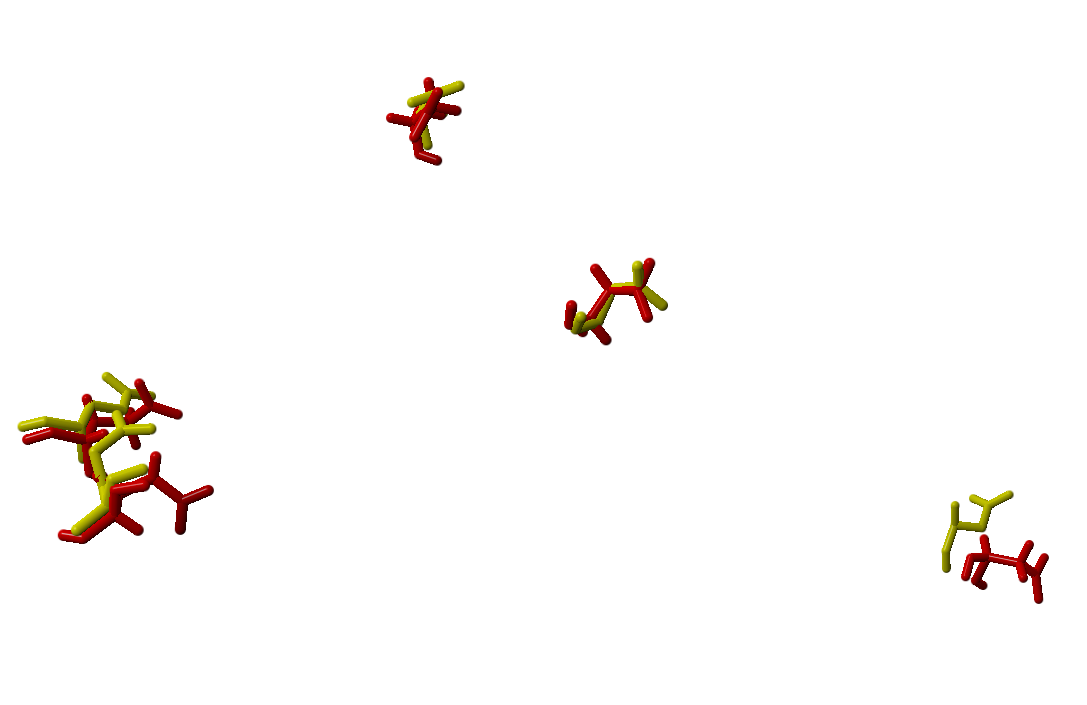


**A**

**B**

**Cab1**

**Cab2**

**Cab3**

**Cab1**

**Cab2**

**Cab3**

**Supplementary Figure 8:** A structural alignment of the crystal structure of Calcium bound microbial Cysteine protease (PDB ID: 4fgo) and the core domain of FXIII-A subunit (PDB ID: 1f13; Residues 183-515). The structures show a RMSD of 1.73 Å with 4 antiparallel beta sheets and one helix conserved between the two structures. The calcium binding sites of the two structures are marked and are clearly spatially differently located.


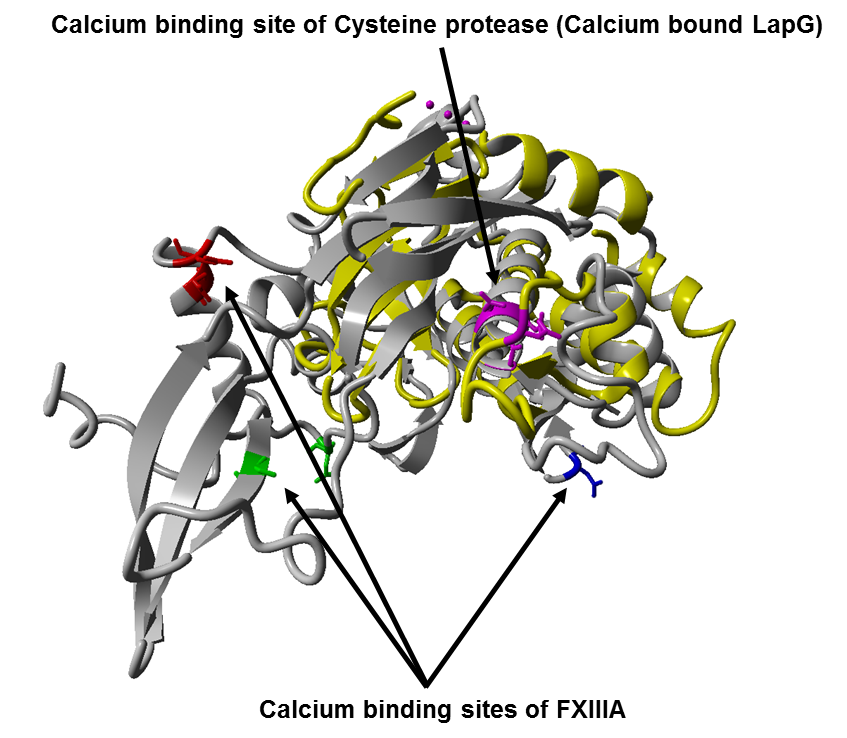


**Supplementary Figure 9a:** Detailed output for the automated modeling for **transglutaminase 1 (TG1)** performed on the *ITASSER* server. Only **Model 1** with the best C-score was considered.
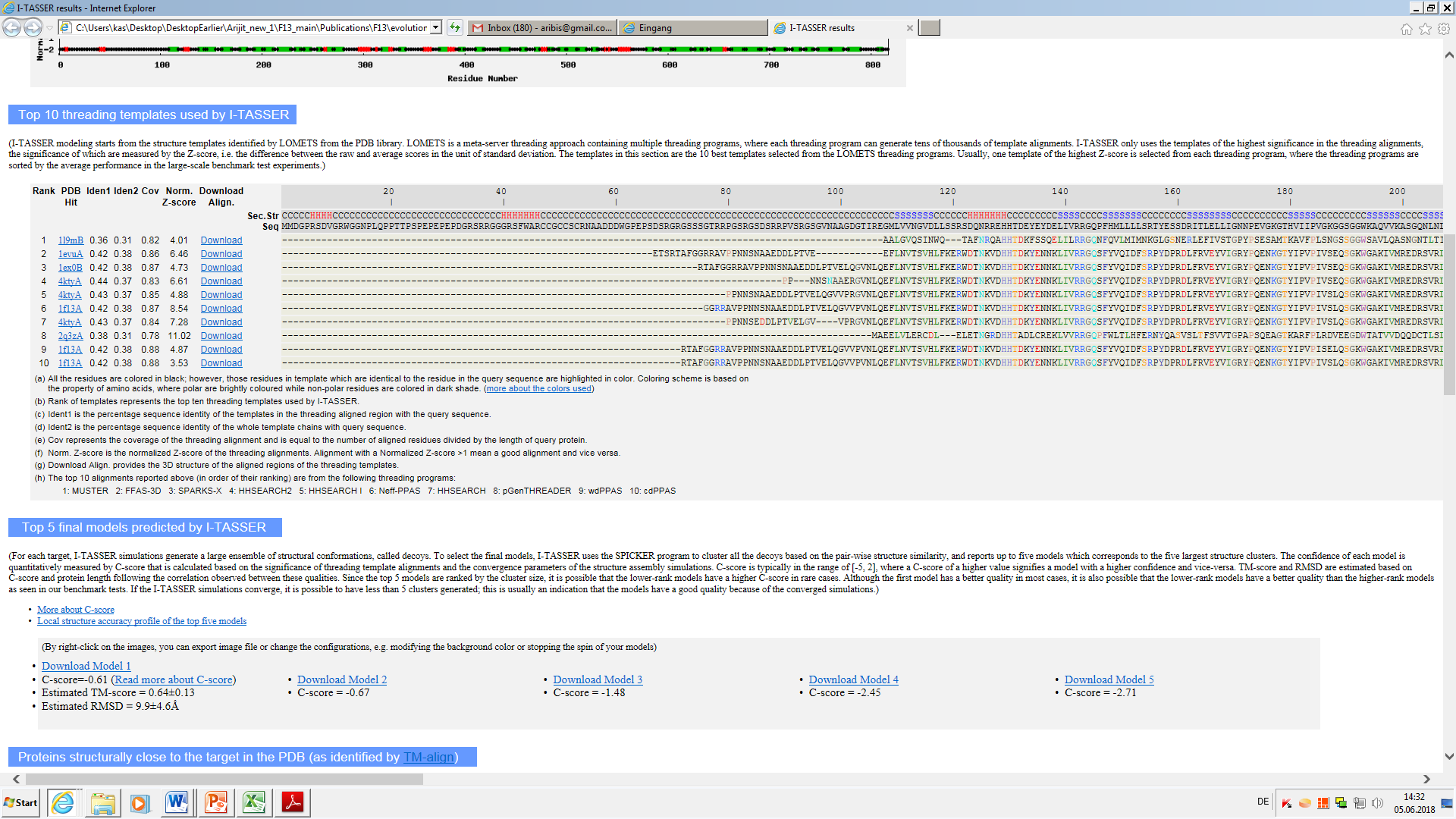


**Supplementary Figure 9b:** Detailed output for the automated modeling for **transglutaminase 4 (TG4)** performed on the *ITASSER* server. Only **Model 1** with the best C-score was considered.


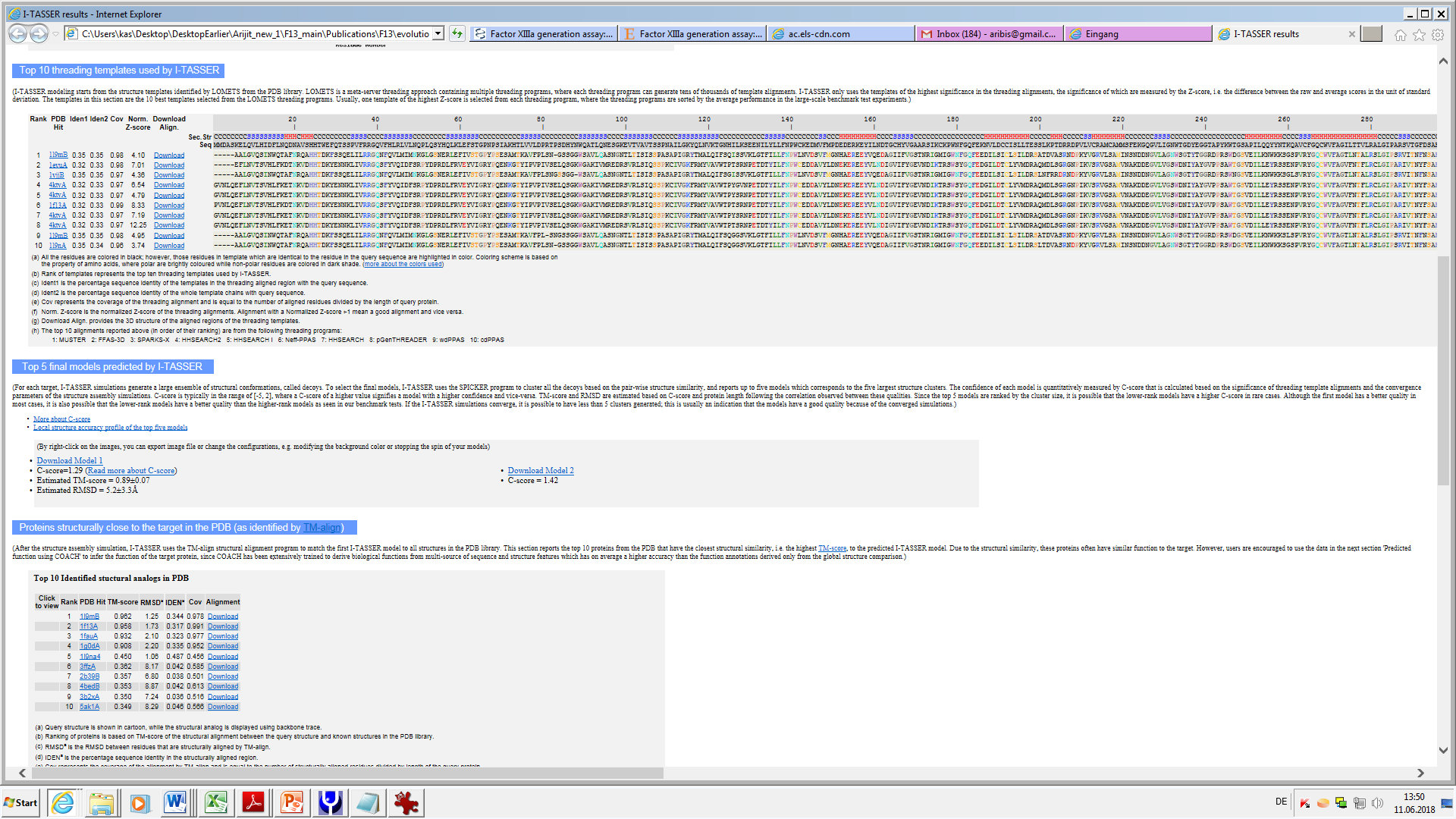


**Supplementary Figure 9c:** Detailed output for the automated modeling for **transglutaminase 6 (TG6)** performed on the *ITASSER* server. Only **Model 1** with the best C-score was considered.


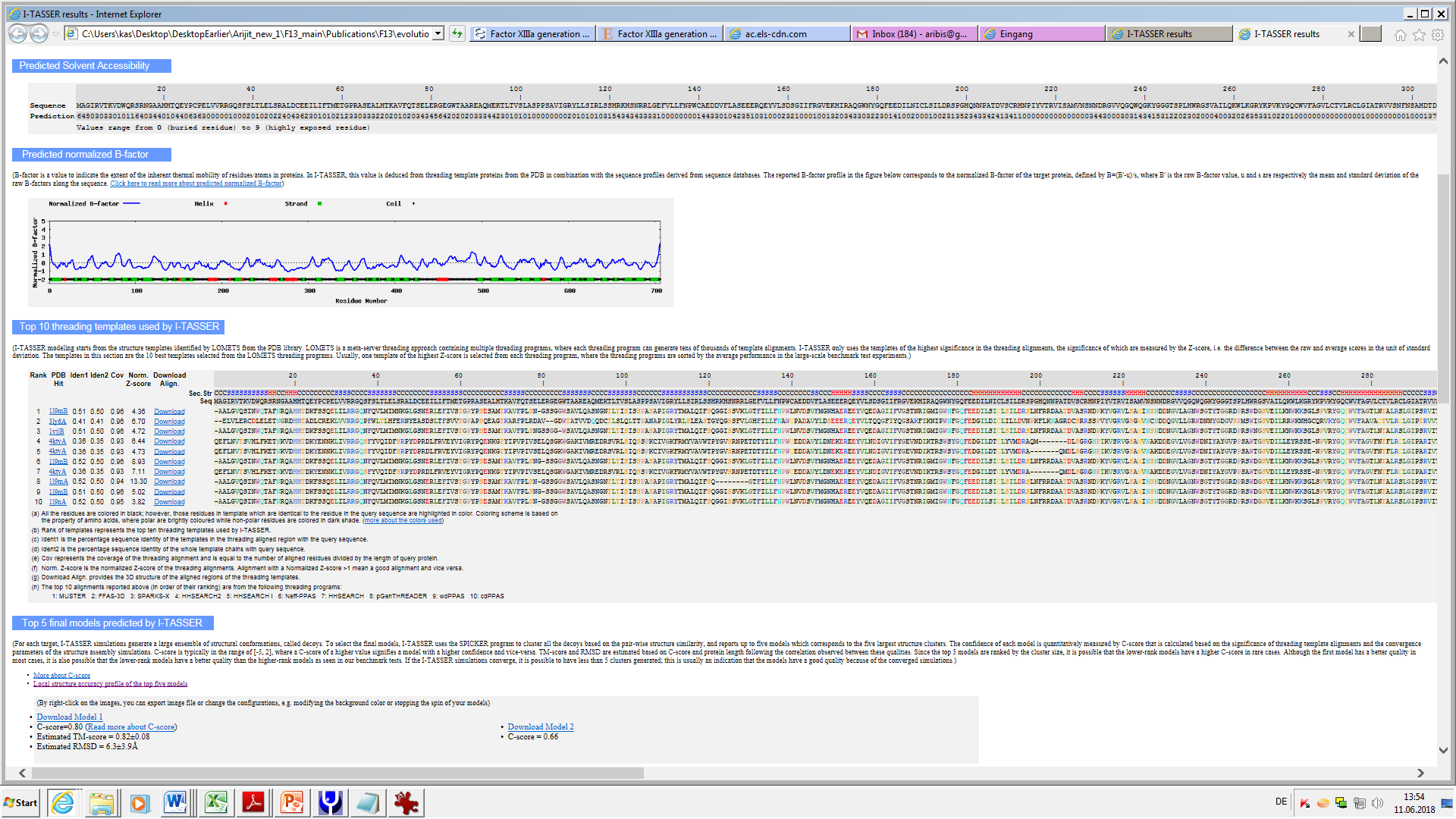


**Supplementary Figure 9d:** Detailed output for the automated modeling for **transglutaminase 7 (TG7)** performed on the *ITASSER* server. Only **Model 1** with the best C-score was considered.


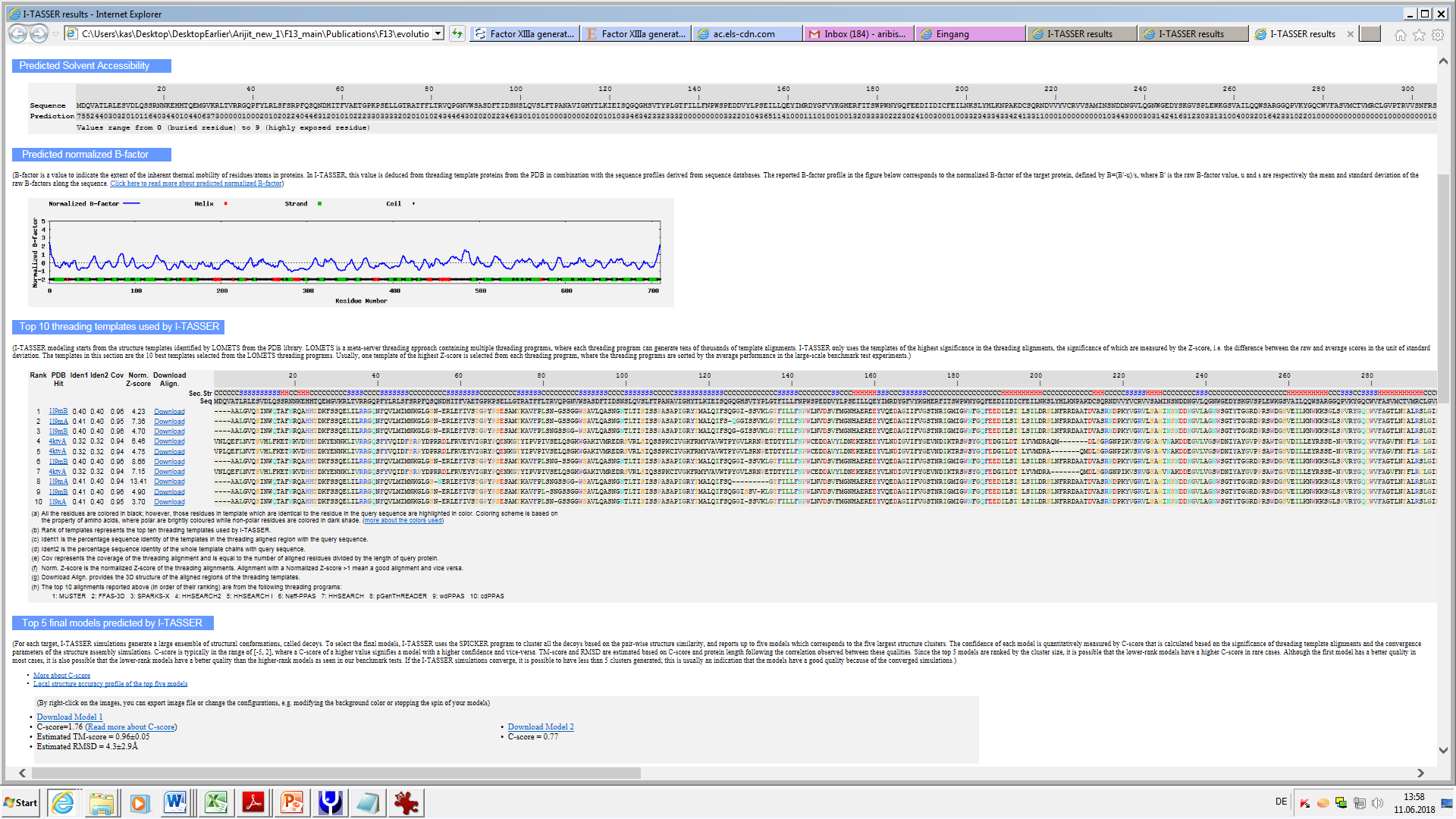


1. * Corresponding author. PD Dr Arijit Biswas Tel.: +4922828419428; Fax: +4922828716087; e-mail: [arijit.biswas@ukbonn.de](mailto:arijit.biswas@ukbonn.de) [↑](#footnote-ref-1)
